# Tumor-suppressive activities of SA1/STAG2 and effects of PARP impairment during brain development

**DOI:** 10.1242/dmm.052440

**Published:** 2026-02-02

**Authors:** Simona Totaro, Antonella Lettieri, Silvia Castiglioni, Francesco Lavezzari, Cristina Gervasini, Valentina Massa, Thomas Vaccari

**Affiliations:** ^1^ Department of Biosciences, Università degli Studi di Milano, 20133 Milan, Italy; ^2^ Department of Health Sciences, Università degli Studi di Milano, 20142 Milan, Italy; ^3^ 'Aldo Ravelli' Center for Neurotechnology and Experimental Brain Therapeutics, Università degli Studi di Milano, 20142 Milan, Italy

**Keywords:** *Drosophila*, STAG2, Cohesin complex, PARP inhibitor

## Abstract

The cohesin complex performs essential cellular functions including regulation of chromatin organization and DNA repair. Somatic pathogenetic variants in cohesin genes, such as *STAG2*, have been associated with cancer, but their contribution to brain tumorigenesis is unclear. Here, we report the presence of *STAG2* variants in patients with glioblastoma and medulloblastoma and determined the effects of loss of *STAG2* in human cells and of the homolog *SA1* in *Drosophila* tissues. Reduction of *SA1* expression during fly brain development led to defects in neural stem cell differentiation and promotion of tumorigenesis, both in the presence and absence of oncogenic activity. Treatment with inhibitors of poly ADP-ribose polymerase (PARP), which are used to treat forms of cancer with defects in DNA repair, in combination with *STAG2/SA1* depletion resulted in apoptosis *in vitro* and *in vivo*. In flies, reduction of PARP activity ameliorated the tumor-associated phenotypes of *SA1-*deficient tissue. Our *in vivo* and *in vitro* data suggest that impairment of PARP activity compensates for reduced cohesin activity, highlighting a vulnerability that could be pharmacologically exploited in brain tumors.

## Teaser

**Summary:** In *Drosophila melanogaster* and in human cells, SA1/STAG2 acts as a tumor suppressor and PARP inhibition reverts the tumor-associated effects of reducing SA1/STAG2 expression during brain development and tumorigenesis.

## INTRODUCTION

Cohesin proteins are part of a conserved ring complex that ensures sister chromatid cohesion (Nasmyth, 2011). The cohesin complex also regulates genomic stability by taking part in DNA repair (Nasmyth and Haering, 2009). In addition, it orchestrates gene expression by acting on the genome 3D architecture and, therefore, by participating in chromatin remodeling (Nasmyth and Haering, 2009). While cohesin genes are essential for survival, heterozygous germline loss-of-function variants cause congenital disorders known as cohesinopathies, including Cornelia de Lange and Roberts syndromes (Kline et al., 2018).

In humans, the cohesin core complex is formed by SMC1A, SMC3, RAD21 and STAG1, STAG2 or STAG3 (Nasmyth and Haering, 2009). While STAG3 is essential for proper chromosome pairing and segregation in meiosis (Bayés et al., 2001), STAG1 and STAG2 have broader, partially overlapping functions. However, STAG2 is specifically required for transcriptional regulation of DNA repair (Romero-Pérez et al., 2019).

Owing to the many tumor suppressive processes in which the cohesin complex is involved, somatic pathogenic variants in multiple cohesin genes were found in several types of tumors (Di Nardo et al., 2022). In particular, *STAG2* is a frequent target of inactivating mutations in human cancers (Hill et al., 2016; Arruda et al., 2020)*.* These usually include frameshift, nonsense or splice site mutations leading to aberrant proteins (De Koninck and Losada, 2016). *STAG2* variants were identified in a variety of tumors, including glioblastoma (Solomon et al., 2011), urothelial bladder cancer (Balbás-Martínez et al., 2013; Guo et al., 2013; Solomon et al., 2013; Taylor et al., 2014; Tirode et al., 2014; The Cancer Genome Atlas Research Network, 2014), melanoma (Solomon et al., 2011), myelodysplastic syndrome (Han et al., 2024), acute myeloid leukemia (Solomon et al., 2011) and Ewing's sarcoma (Brohl et al., 2014; Crompton et al., 2014). Somatic variants in *STAG1* are also involved in the tumorigenesis of colorectal cancer, bladder cancer, Ewing's sarcoma and myeloid malignancies (Balbás-Martínez et al., 2013; Kon et al., 2013; Thota et al., 2014; The Cancer Genome Atlas Research Network, 2014).

Cohesin genes are functionally conserved in *Drosophila melanogaster.* In particular, the fly genome encodes two homologs of human *STAG1-3*. *Stromalin 1* (*SA1*; CG3423) is ubiquitously expressed and appears to be related to *STAG1/2*, while *Stromalin 2* (*SA2*; CG13916) is mainly expressed in male gonads and likely to be functionally similar to *STAG3* (Thomas et al., 2005)*. SA1* was initially identified with other fly cohesin genes for its ability to bind chromatin and regulate enhancer–promoter communication and support sister chromatid cohesion (Rollins et al., 2004; Gause et al., 2008). During embryogenesis, *SA1* expression is controlled by the Notch target Cut in neuroblasts (NBs) and supports developmentally regulated NB death, preventing the emergence of ectopic NBs. Such a process appears to depend on cohesin’s ability to regulate chromatin architecture (Arya et al., 2019). In the developing larval fly brain, *SA1* has also been implicated in post-mitotic regulation of morphogenesis, in particular in neuronal pruning, a process dependent on transcriptional regulation (Schuldiner et al., 2008), as well as in establishing the pool of synaptic vesicles for memory formation (Phan et al., 2019). *SA1* contribution to tumor biology has not yet been assessed in flies. Despite this, a number of genetic manipulations in *Drosophila* have previously been used to assess the contribution of specific genes to brain tumorigenesis (Read et al., 2009; Paglia et al., 2017; Maurange, 2020).

Poly ADP-ribose polymerase (PARP) is a central sensor of DNA damage (D'Andrea, 2018). Drugs that inhibit PARP activity are currently used to induce synthetic lethality of breast and ovarian cancer cells with variants in genes regulating DNA repair pathways (D'Andrea, 2018; Mekonnen et al., 2022). A few lines of evidence in *in vitro* systems have reported that depletion of *STAG2* causes susceptibility to PARP inhibitors (Bailey et al., 2014; Liu et al., 2018; Mondal et al., 2019; Zhou et al., 2023; Luo et al., 2024). While the first evidence of *STAG2* involvement in tumorigenesis was the presence of focal deletions on the X chromosome in glioblastoma (Solomon et al., 2011), no animal models of brain tumorigenesis based on reduced *STAG1/2* in somatic cells exist, and no study of the effects of PARP inhibitors *in vivo* has been reported.

Here, we updated the repertoire of *STAG2* variants associated with glioblastoma and medulloblastoma. We found that *STAG2* depletion in human spheroids leads to persistent DNA damage and that treatment with a PARP inhibitor increased the amount of apoptosis, compared to that in untreated samples. To model the effect of somatic *STAG2* deficiency *in vivo*, we reduced the expression of the *Drosophila* homolog *SA1* in multiple tissues during development. In larval wing disc, reduced expression of *SA1* and impairment of the activity of *Parp1*, the unique homolog of human genes encoding PARP enzymes, led to additive stabilization of DNA damage and to caspase activation. These phenotypes correlate with amelioration of the adult wing morphology, compared to that obtained with single manipulation. In a larval model of gliomagenesis, we found that reduced *SA1* expression causes excess tissue growth, which is prevented by reduction in *Parp1* expression. Finally, in larval type II NBs (NBIIs), a type of neural stem cell (NSC) that gives rise to neurons and glia, upon *SA1* expression reduction, we observed a delay of differentiation that is accompanied by occasional formation of masses in adult brains and shortened lifespan. *Parp1* depletion reverted *SA1* NBII phenotypes. Our data indicate that cohesin genes act as tumor suppressors and that their loss can be compensated by PARP inactivation *in vivo*. Thus, brain tumors with cohesin variants might represent potential therapeutic targets for PARP inhibitors.

## RESULTS

### *STAG2* variants are present in patients with glioblastoma and medulloblastoma

**Fig. 1.**
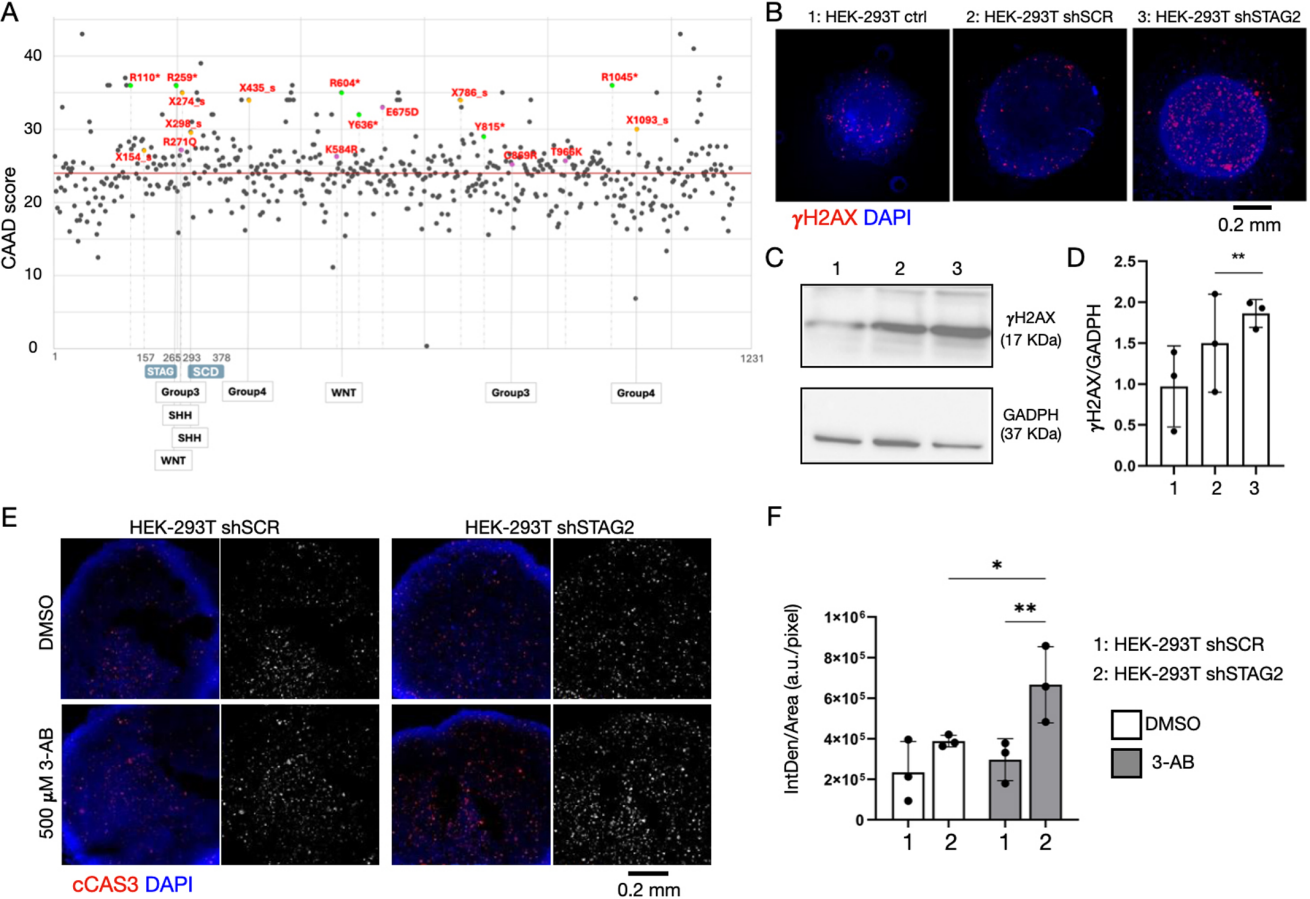
**STAG2 variants in brain cancer, *STAG2* depletion and PARP inhibitor treatment in human cells.** (A) Variants in STAG2 observed in medulloblastoma and glioblastoma are deleterious. The graph displays the CADD value (*y*-axis), a score of variant deleteriousness, for all the STAG2 variants reported in the genome aggregation database gnomAD (gray) and in the cancer genomic database cBioPortal (in color). Missense variants associated with medulloblastoma and glioblastoma are shown as colored dots. Purple indicates missense, green nonsense and yellow splicing variants. Three frameshift variants found in medulloblastoma and glioblastoma are not reported. Gray variants are mainly found in Mulleghama–Klein–Martinez syndrome patients. The position of the amino acid (aa) changes is shown on the *x*-axis and above the medulloblastoma and glioblastoma variants. The average CADD value of variants is indicated by the red line. Below the graph, labels indicate the molecular subtyping of medulloblastoma for the indicated variants. SCD, stromal in conserved domain. (B) Confocal sections of control HEK-293T spheroids or spheroids stably expressing a scrambled control shRNA (shSCR) or a shRNA targeting *STAG2* (shSTAG2) treated to label DNA (DAPI) and DNA damage foci (γH2AX). (C,D) Western blot analysis of the indicated extracts to detect γH2AX and GADPH levels (C) and relative quantification (D). In D, each data point represents a biological replicate. HEK-293T shSTAG2 samples were compared to HEK-293T shSCR by one-way ANOVA, with uncorrected Dunnett's multiple comparison test. **P<0.01. (E,F) Confocal sections of the indicated spheroids treated as indicated and labeled to detect the DNA (DAPI) and the apoptotic marker cleaved caspase 3 (cCas3) (E) and relative quantification (F). In F, each data point represents a biological replicate. The 3-aminobenzamide (3-AB) shSTAG2 sample was compared to 3-AB shSCR or vehicle-treated shSCR by one-way ANOVA, with uncorrected Fisher's LSD test. *P<0.05, **P<0.01. All other comparisons are non-significant. a.u., arbitrary units.

To evaluate the presence of *STAG2* variants in patients diagnosed with glioblastoma multiforme or medulloblastoma, we examined the publicly accessible cBioPortal, selecting datasets from eight studies encompassing 1538 patients. We detected *STAG2* variants in 21 patients (2%) – specifically, five missense, seven nonsense, six splicing and three frameshift variants. Eight were found in patients with medulloblastoma and 13 in patients with glioblastoma (colored dots in Fig. 1A). These changes were distributed throughout the gene, involving the entire amino acid (aa) sequence of STAG2. Despite the lack of mutational hotspots, we observed that four of the eight medulloblastoma STAG2 variants occur within 39 aa corresponding to the end of the stromal antigen (STAG) domain and the beginning of the stromal in conserved domain (SCD). For the medulloblastoma-associated variants, we also observed no association between a particular type of aa change with molecular subtypes (Fig. 1A).

Compared to variants associated with Mulleghama–Klein–Martinez syndrome, a rare X-linked neurodevelopmental disorder caused by variants in *STAG2* (Mullegama et al., 2017), the glioblastoma multiforme or medulloblastoma variants were assigned a Combined Annotation Dependent Depletion (CADD) score (see Materials and Methods for details) well above the average (Fig. 1A), suggesting that they might lead to reduced STAG2 function. Notably, in two patients with medulloblastoma (Group 3 and WNT group), the same variant, R259*, was reported. Patient details are listed in Table S1. The bioinformatic analysis suggests that somatic variants in brain cancer lead to partial or total loss of *STAG2* function.

### Synergism of STAG2 knockdown and PARP inhibition in an *in vitro* model

To test the primary consequences of reduced *STAG2* function *in vitro*, we developed a cell model based on *STAG2* silencing in non-transformed cells. To this end, we stably integrated in HEK-293T cells a lentiviral plasmid expressing a short hairpin against *STAG2* (shSTAG2) or a scrambled sequence (SCR) as a control. To assess *STAG2* knockdown, we evaluated the expression of *STAG2* mRNA and protein. We observed that STAG2 was significantly depleted in HEK-293T shSTAG2, compared with in HEK-293T shSCR and HEK-293T control (Fig. S1A), leading to reduction in protein expression (Fig. S1B,C). Depletion did not cause significant changes in cell viability but influenced cell adhesion to the substrate (Fig. S1D-F), consistent with previously reported cellular phenotypes observed upon *STAG2* depletion (Mondal et al., 2019).

Considering that the cohesin complex is involved in DNA repair, we next prepared 3D HEK-293T cultures and investigated spheroids for the presence of DNA damage. To this end, we analyzed positivity for γH2AX, a well-known DNA damage marker, by western blot analysis. We observed that HEK-293T shSTAG2 spheroids show higher amounts of γH2AX than do HEK-293T shSCR or control spheroids (Fig. 1B-D).

Given the increased DNA damage in STAG2-depleted cells and the reported susceptibility of cells with aberrant DNA repair mechanisms to PARP inhibitors, we assessed the possible effects of PARP inhibition in our *in vitro* model. Thus, we treated HEK-293T shSTAG2 and shSCR with 3-aminobenzamide (3-AB), a potent PARP inhibitor (Sodhi et al., 2010). After 72 h of treatment, to detect the presence of apoptotic cells, we performed a terminal deoxynucleotidyl transferase dUTP nick-end labeling (TUNEL) assay or immunofluorescence for cleaved caspase 3 (cCas3), a marker of apoptosis. We found that HEK-293T shSTAG2 spheroids treated with 3-AB displayed significantly higher cCas3 positivity compared with DMSO-only HEK-293T shSTAG2 spheroids or with 3-AB-treated HEK-293T shSCR spheroids (Fig. 1E, quantified in Fig. 1F). HEK-293T shSTAG2 cells treated with 3-AB also showed significantly higher TUNEL positivity compared with HEK-293T shSTAG2 treated with vehicle or 3-AB-treated HEK-293T shSCR cells (Fig. S1G, quantified in Fig. S1H). Overall, our *in vitro* data suggest the presence of a pharmacogenetic synthetic lethal interaction between the loss of STAG2 and PARP activity, which might depend on DNA damage.

### Reduction of *SA1* activity in *Drosophila melanogaster* affects DNA repair *in vivo*

**Fig. 2.**
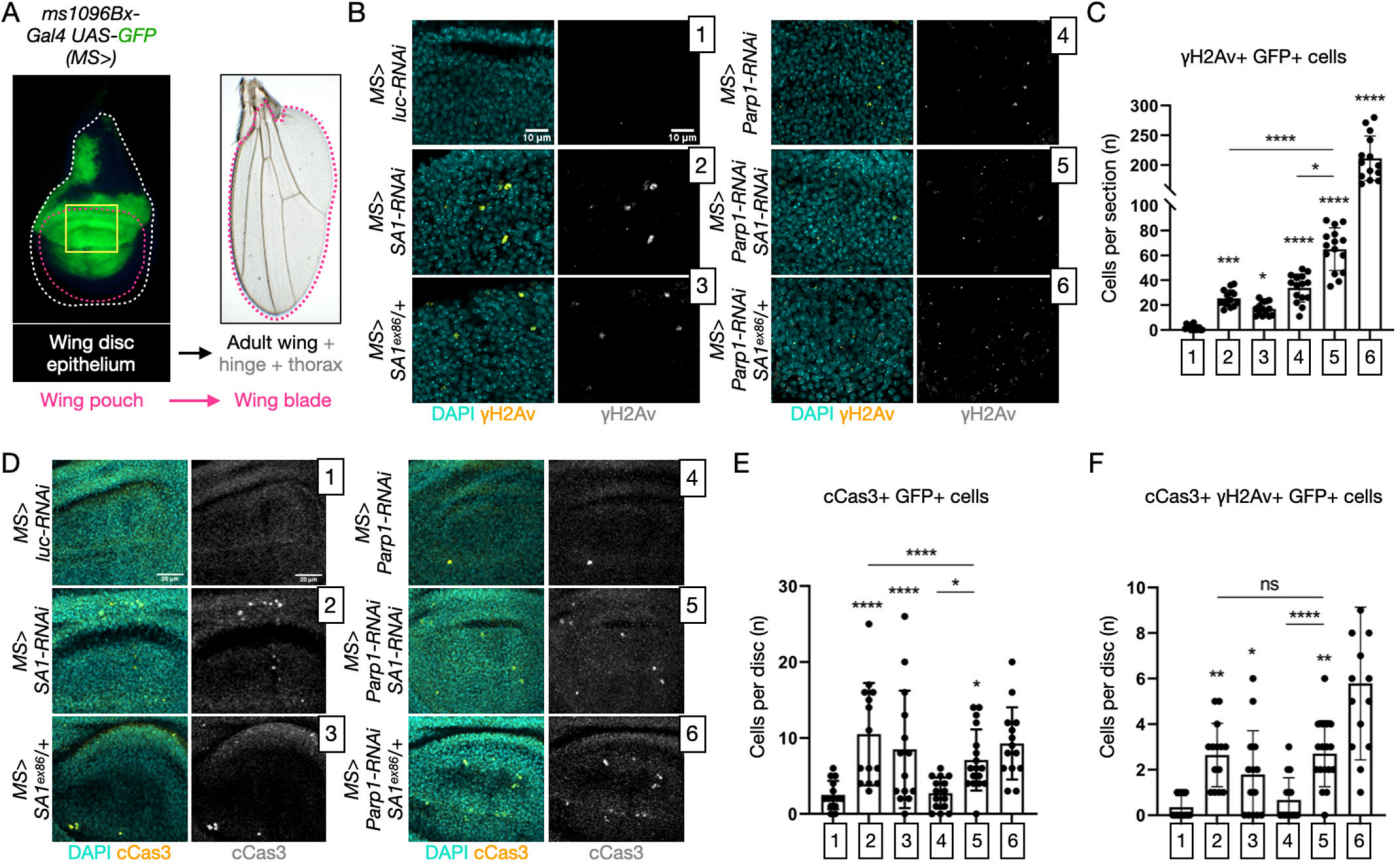
**DNA damage and apoptosis upon *in vivo* downmodulation of cohesin and PARP activity.** (A) Schematics of *Drosophila* wing imaginal discs illustrating their structure, fate and domain of misexpression using *MS-Gal4 UAS-GFP* (*MS>*) to mark the tissue impacted by the depletions. The area analyzed in the experiment described in this figure is highlighted by the yellow box. (B,C) Single confocal imaging sections of the GFP^+^ dorsal portion of the wing pouch depleted as indicated and treated to label DNA (DAPI) and DNA damage foci (γH2Av) (B) and relative quantification (C). Sections were analyzed to count the number of cells with γH2Av signal. Samples were compared by one-way ANOVA, with uncorrected Fisher's LSD. *P<0.05, ***P<0.001, ****P<0.0001. *P*-value intervals above the bars indicate comparisons with sample 1; lines indicate other meaningful comparisons. Each data point in C represents a single disc section. *N*>14 sections analyzed. (D-F) Confocal sections of the pouch of imaginal discs depleted *in vivo* as indicated, treated to label cleaved Caspase 3 (cCas3) (D), and relative quantification (E,F). *Z*-stacks of the GFP^+^ portion of the wing discs were scored to count the average number of cells with cCas3 signal. Samples were compared by one-way ANOVA, with uncorrected Fisher's LSD. Non-significant (ns), *P*>0.05; *P<0.05, ****P<0.0001. *P*-value intervals above the bars indicate comparisons with sample 1; lines indicate other meaningful comparisons. Each data point in E,F represents a *z*-stack. *N*>14 sections analyzed.

To validate our *in vitro* results, we studied the consequences of somatic depletion of *Drosophila SA1* and *Parp1 in vivo*. To deplete *SA1*, we expressed TRiP.GL00534 or TRiP.HMS00272, two hairpins to induce RNA interference (RNAi) against *SA1.* To reduce expression in cells of the dorsal side of larval wing imaginal discs, a monolayer epithelial tissue that gives rise to the adult wing, we used the driver *ms1096Bx-Gal4*. We co-expressed UAS-GFP to mark the tissue impacted by the downregulations (Fig. 2A). Compared to mock downregulation of luciferase (*MS>luc-RNAi*), TRiP.GL00534 (SA1-RNAi hereafter) was slightly more efficient than TRiP.HMS00272 in downregulating *SA1* expression levels (Fig. S2A). Thus, we selected it for further analyses. *Parp1* depletion, achieved by expression of the GD9445 RNAi hairpin (*MS>Parp1-RNAi*), caused nucleolar fragmentation, as previously reported (Boamah et al., 2012), indicating that it efficiently inactivates Parp1 (Fig. S2B, quantified in Fig. S2D). Supplementation of 3-AB in the fly food also caused nucleolar fragmentation in epithelial tissue (Fig. S2C, quantified in Fig. S2D), indicating that the inhibitor is bioactive and was well tolerated (Fig. S2E).

Having established that our pharmacogenetic manipulations are effective in wing disc cells, we proceeded to immunolocalize DNA damage foci using an antibody against the marker γH2Av (also known as His2av) and apoptotic cells with an antibody against the marker cCas3. Although control *MS>luc-RNAi* cells presented no DNA damage foci, we observed that GFP^+^ cells of *MS>SA1-RNAi* animals displayed a significant amount of DNA damage. Similar results were obtained in *MS>Parp1-RNAi* animals, or in *SA1^ex86^/+* animals, which are heterozygous for a *SA1* loss-of-function allele, indicating that the phenotype is not due to off-target effects (Fig. 2B, quantified in Fig. 2C). Interestingly, *Parp1-RNAi* GFP^+^ cells that are also depleted or heterozygous for *SA1* display more DNA damage foci, compared to cells with single manipulations (Fig. 2B, quantified in Fig. 2C). These data suggest that combined reduction of *SA1* and *Parp1* leads to an additive effect on DNA damage *in vivo*.

To determine whether the observed DNA damage is associated with Caspase-dependent apoptotic cell death, we also quantified the GFP^+^ cCas3^+^ cells. Whereas control *MS>luc-RNAi* or *MS>Parp1-RNAi* cells presented only occasional cCas3 staining, we observed that *MS>SA1-RNAi* or *SA1^ex86^/+* cells displayed a significant amount of cCas3 signal (Fig. 2D, quantified in Fig. 2E). These data suggest that reduced *SA1* expression, but not reduced *Parp1* expression, causes Caspase-dependent apoptotic cell death. Consistent with this, when combining reduced *SA1* and *Parp1* expression, no additional Cas3^+^ signal was observed *in vivo* (Fig. 2D, quantified in Fig. 2E). A similar pattern was observed upon quantification of the few cells per disc that are double positive for DNA damage foci and cCas3 expression (Fig. 2F). Together, these data suggest that cells with reduced *SA1* and *Parp1* expression present high level of DNA damage and that a certain proportion of cells with reduced *SA1* expression are likely to be eliminated by Caspase-dependent apoptosis.

Consistent with the alterations observed in the larval wing pouch, *MS>SA1-RNAi* adult animals displayed curled wings, some of which with blisters (Fig. S3A, quantified in Fig. S3B). Compared with *MS>SA1-RNAi* animals, *MS>SA1-RNAi, Parp1* animals displayed a slight statistically significant reduction in the frequency of animals with blisters (Fig. S3B). Slight amelioration of the wing phenotype was also observed upon feeding 3-AB to *MS>SA1-RNAi* animals (Fig. S3C). Wings appeared normal in *MS>luc-RNAi* animals, *SA1^ex86^/+* animals, *MS>Parp1-RNAi* animals and *MS>Parp1-RNAi, SA1^ex86^/+* animals, or in *MS>luc-RNAi* animals that had been fed 3-AB (Fig. S3A,B), indicating that Parp1 inactivation or halving *SA1* dosage do not affect wing development, either alone or in combination. These *in vivo* data suggest that reduction of *Parp1* modifies the defects of *SA1-*depleted animals.

### Cohesin activity is tumor suppressive during gliomagenesis

**Fig. 3.**
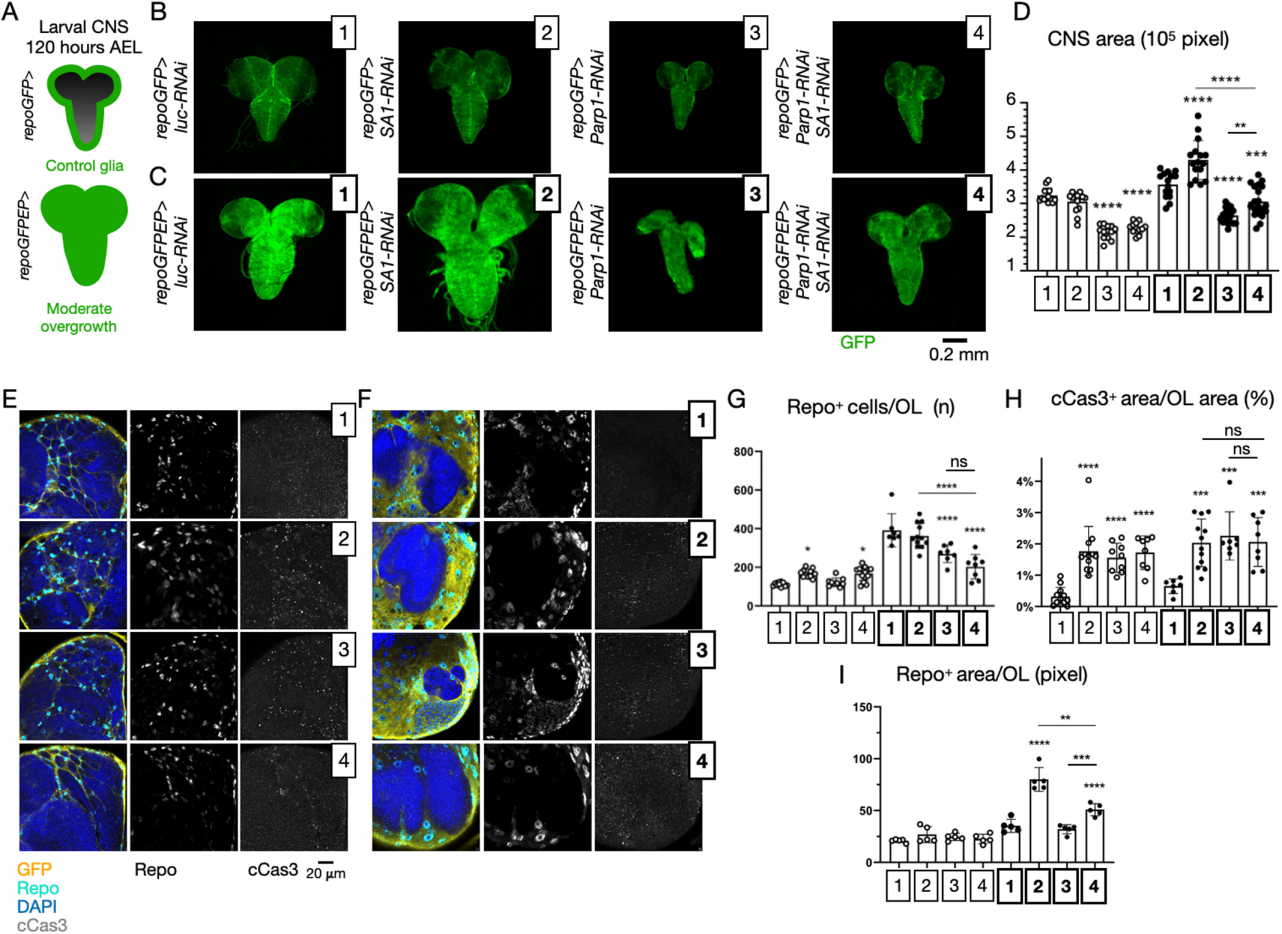
**Tumor-suppressive activity of cohesin genes in an *in vivo* model of glioma.** (A) Schematics of the gliomagenesis model in *Drosophila* larvae. *repoGFPEP* larvae at 120 h after egg laying (AEL) display a moderate overgrowth that constitutes a useful sensitized background to evaluate the effects of genetic modulations. (B-D) Maximum projections of confocal *z*-stacks spanning the entire larval central nervous system (CNS) of animals of the indicated genotype (B,C) and relative quantification of the CNS area (D). The area of >14 CNSs per sample is indicated. Samples were compared by one-way ANOVA, with Šídák's multiple comparisons test. **P<0.01, ***P<0.001, ****P<0.0001. *P*-value intervals above the bars indicate comparisons with sample 1; lines indicate other meaningful comparisons. (E-I) Single medial sections of the CNS labeled as indicated (E,F) and associated quantifications (G-I). Genotypes are as in B,C. In G,H, each data point represents the number of Repo^+^ cells per optic lobe (OL) (G), or the percentage of the cCas3^+^ signal over the optic lobe area. *N*>7 optic lobes per sample were analyzed. In I, each data point represents the mean area of 20 cells. *N*=5 optic lobes per sample. Samples in G-I were compared by one-way ANOVA, with Šídák's multiple comparisons test. Non-significant (ns), *P*>0.05; **P<0.01, ***P<0.001, ****P<0.0001. *P*-value intervals above the bars indicate comparisons with sample 1; lines indicate other meaningful comparisons.

To assess the contribution of cohesin genes to brain tumorigenesis *in vivo*, we employed a genetic model of gliomagenesis based on expression in developing larval glial cells of constitutively active forms of *Drosophila* Egfr and of the PI3K homolog Pi3K92E/Dp110 (Read et al., 2009), and of mC8GFP to mark the glial tissue (Fig. 3A; *repoGFPEP>* hereafter). To assess the effects of cohesin activity on glia cell development independent of tumorigenesis, we first depleted five cohesin genes – *SA1*, *SMC1*, *SMC2*, *Nipped-B*, *vtd* and *Mau2* – individually in control glia expressing mCD8::GFP (*repoGFP>* hereafter). Compared to expression of a mock hairpin targeting *luciferase* (*repoGFP>luc-RNAi*), we did not observe major changes in central nervous system (CNS) tissue growth at 120 h after egg laying (AEL), with the exception of depletion of *Mau2*, which slightly reduced growth (Fig. S4A, quantified in Fig. S4B). Compared to *repoGFP>luc-RNAi* animals, *repoGFPEP>luc-RNAi* animals at 120 h AEL displayed a moderate CNS overgrowth that could be used as a sensitized background to evaluate the effect of further genetic modulations (Fig. S4C, quantified in Fig. S4B). Interestingly, depletion of the cohesin genes *SA1*, *SMC1* and *SMC3* in *repoGFPEP* animals led to increased CNS size and caused earlier lethality, compared to that in *repoGFPEP>luc-RNAi* animals (Fig. S4C, quantified in Fig. S4B; Fig. S5).

We next assessed the effects of Parp1 depletion on the phenotypes described above. Relative to control *repoGFP>luc-RNAi* and to *repoGFP>SA1-RNAi* animals, *repoGFP>Parp1-RNAi* or *repoGFP>Parp1-RNAi SA1-RNAi* animals displayed reduced CNS size, indicating that Parp1 is required to support CNS growth (Fig. 3B, quantified in Fig. 3D). Remarkably, a similar reduction in CNS size was observed in *repoGFPEP* animals, indicating that *Parp1* inactivation can fully revert the effects of reduced SA1 activity (Fig. 3C, quantified in Fig. 3D).

To investigate the origin of the effects described above, we labeled the nuclei of the glial cells with an antibody against Repo, and the cells undergoing Caspase-dependent apoptosis with anti-cCas3 (Fig. 3E-I). In control glia, we observed that depletion of *SA1* slightly increased the number of glial nuclei (Fig. 3E, quantified in Fig. 3G), while depletion of *SA1*, *Parp1* or both, led to recovery of a significant amount of cCas3^+^ cells (Fig. 3E, quantified in Fig. 3H). Similar recovery of cCas3^+^ cells was observed in the CNS of animals bearing glial tumors (Fig. 3F, quantified in Fig. 3H). In glial tumors, depletion of *SA1* did not cause an increase in the number of Repo^+^ nuclei (Fig. 3F, quantified in Fig. 3G), but their nuclear size was found to be increased (Fig. 3F, quantified in Fig. 3I). Interestingly, *Parp1* depletion reduced the nuclear size of *SA1*-depleted glial cells, compared to that of *SA1*-depleted glial cells (Fig. 3F, quantified in Fig. 3I).

Overall, these data suggest that some cohesin genes, among which *SA1*, act as tumor suppressors and that *Parp1* activity is required to support the tissue growth caused by reduction in *SA1* expression. These effects appear to correlate with glial nuclear size (compare Fig. S3D with Fig. S3I), rather than glial cell number or apoptosis, possibly suggesting an effect on cell size.

### *SA1* depletion during development leads to retention of undifferentiated cell masses in the adult brain

**Fig. 4.**
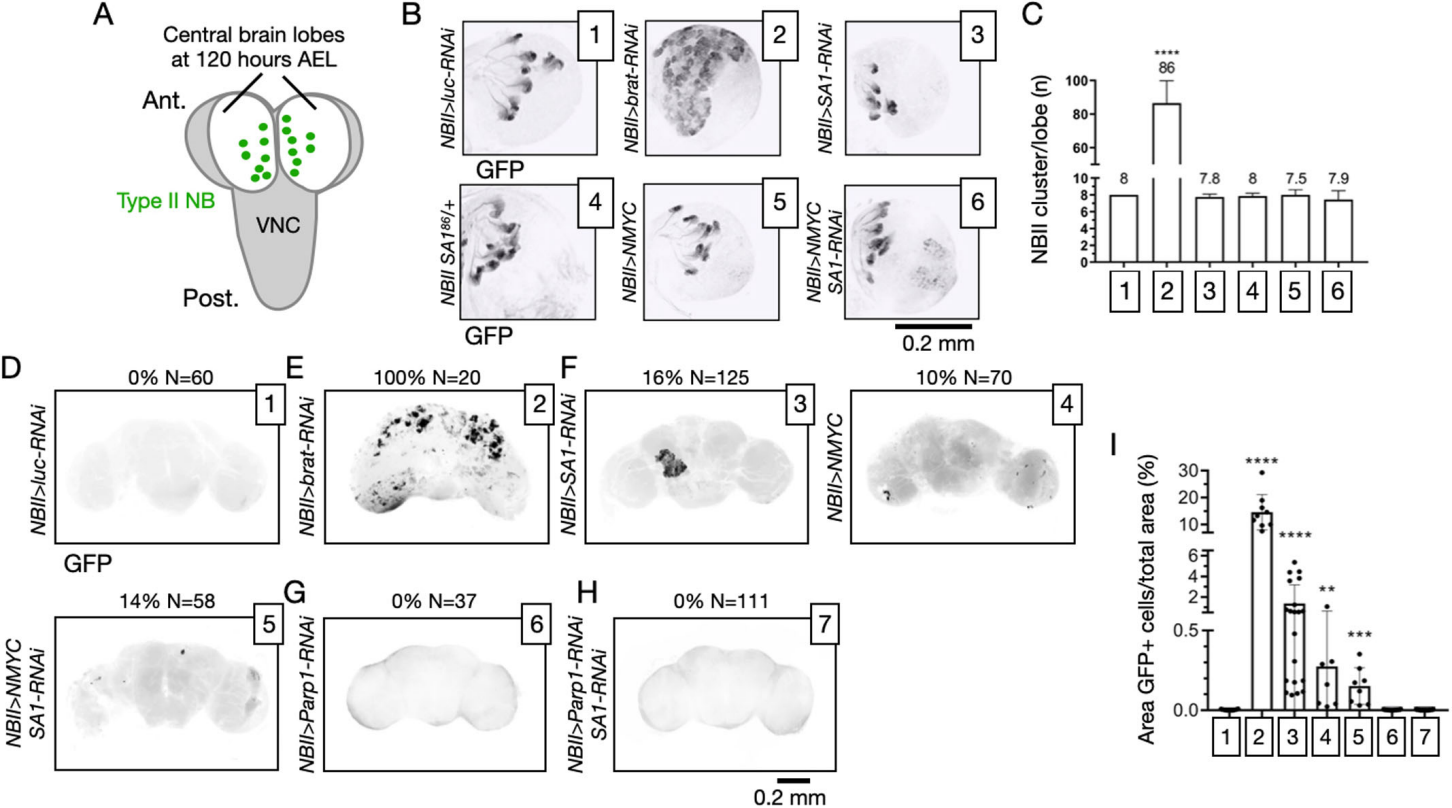
**Formation of masses upon manipulation of oncogenes and *SA1* expression in *Drosophila* neural stem cells.** (A) Schematics of neuroblast (NB) positioning in the larval CNS at 120 h AEL. VNC, ventral nerve cord. (B) Maximum projections of confocal *z*-stacks spanning optic lobes of the indicated genotypes showing GFP expression associated with larval type II NBs (NBIIs). (C) Quantification of NBII number per optic lobe upon modulation of the indicated genes. *N*>6 optic lobes per sample. Samples were compared by one-way ANOVA, with Šídák's multiple comparisons test. ****P<0.0001. *P*-value intervals above the bars indicate comparisons with sample 1. (D-H) Maximum projections of confocal *z*-stacks spanning adult brains of the indicated genotypes showing GFP signal or lack of thereof. (I) Quantification of the area of the brain covered by GFP^+^ masses. Samples as in D-H. Each data point represents one brain positive for GFP. *N*>7 brains analyzed. Samples were compared by uncorrected Kruskal–Wallis test. **P<0.01, ***P<0.001, ****P<0.0001. *P*-value intervals above the bars indicate comparisons with sample 1.

To further analyze the role of *SA1* in brain tumorigenesis, we used a genetic background that drives gene expression in larval NBIIs (Neumüller et al., 2011), the eight NSCs that generate neurons and glia of the posterior part of each hemisphere of the larval fly brain (Fig. 4A). In control animals at 120 AEL, NBIIs drove expression of mCD8::GFP to mark the NBII lineage and of a mock hairpin targeting *luciferase* (*NBII>luc-RNAi*). Depletion of the tumor suppressor *brat* (*NBII>brat-RNAi*) led to a massive increase in the number of NBII clusters. In contrast, expression of the human brain tumor oncogene *NMYC* (also known as *MYCN*; *NBII>NMYC*), *SA1* heterozygosity (*NBII SA1^86^/+*) or depletion of *SA1* (*NBII>SA1-RNAi*), or the combination of the two manipulations (*NBII>NMYC SA1-RNAi*), did not significantly alter NBII numbers (Fig. 4B, quantified in Fig. 4C), indicating that *SA1* reduction does not increase NBII numbers.

To visualize dividing cells in NBII clusters, we immunolabeled with anti-phospho-Histone H3 (pHis3). We did not observe a large variation in the number of proliferating cells upon depletion of *brat*, or overexpression of NMYC, while depletion of SA1 significantly reduced the number of proliferating cells (Fig. S6A, quantified in Fig. S6B). In *NBII>NMYC*, *NBII>NMYC SA1-RNAi* or *NBII>SA1-RNAi* animals, we occasionally observed the formation of aberrant mitotic figures, a defect not observed in control *NBII>luc-RNAi* animals or *NBII>brat-RNAi* animals (Fig. S6A). These data suggest that *SA1* depletion does not promote proliferation of NBIIs.

NBII expression is known to abate with full maturation of NB clusters at the end of pupal life. In agreement with this, we never recovered GFP^+^ cells persisting in the adult brain of *NBII>luc-RNAi* animals (Fig. 4D, quantified in Fig. 4I). Thus, we wondered whether our manipulations could lead to persistence of GFP^+^ cells in the brain, as previously reported for tumorigenesis mediated by loss of *brat* (Hadjipanayis and Brat, 2017; Reichardt et al., 2018). Consistent with this, all *NBII>brat-RNAi* animals presented masses of GFP^+^ cells covering a large part of their adult brain (Fig. 4E, quantified in Fig. 4I). Interestingly, the occasional presence of GFP^+^ cells covering a small and highly variable part of the adult brain was also observed in *NBII>NMYC*, *NBII>NMYC SA1-RNAi* or *NBII>SA1-RNAi* animals (Fig. 4F, quantified in Fig. 4I). In adult brains, GFP^+^ masses were never recovered in *NBII>Parp1-RNAi* animals (Fig. 4G, quantified in Fig. 4I). The same result was observed by co-depletion of *Parp1* and *SA1* (Fig. 4H, quantified in Fig. 4I), suggesting that Parp1 activity is required for persistence of the masses.

To test the levels of apoptosis upon *SA1* and *Parp1* depletion in NBIIs, we compared animals at 120 AEL. We observed that, in control *NBII>luc-RNAi* animals, no cCas3^+^ cell was ever recovered (Fig. S6C). In contrast, a variable proportion of cCas3^+^ cells was recovered in *NBII>Parp1-RNAi*, *NBII>SA1-RNAi* or *NBII>Parp1-RNAi SA1-RNAi* clusters (Fig. S6C).

Overall, these data indicate that reduction of *Parp1* activity prevents the formation of masses caused by reduced *SA1* expression, possibly by apoptotic elimination.

### PARP inhibition improves NB differentiation and lifespan in *SA1* knockdown flies

**Fig. 5.**
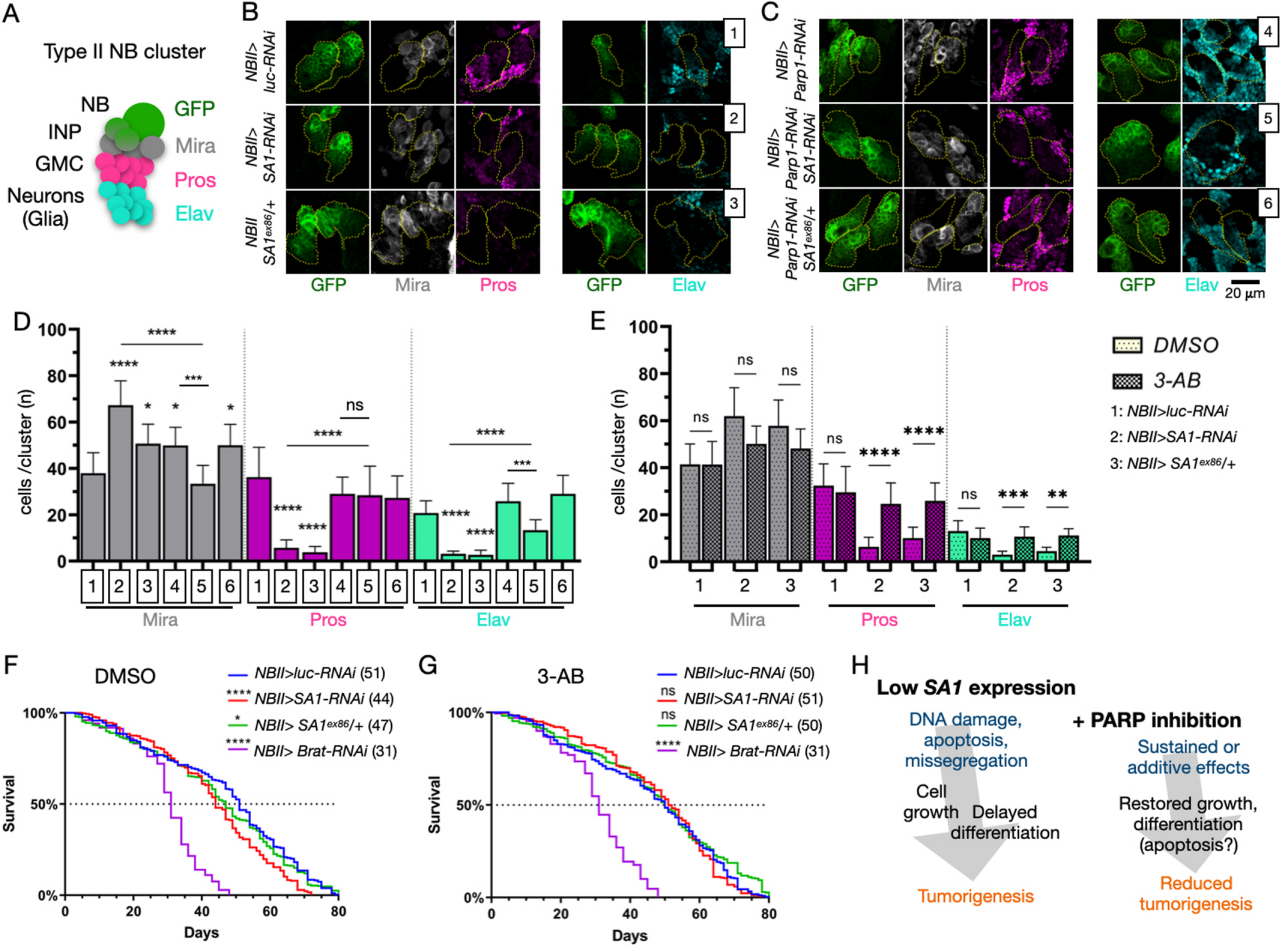
**Developmental and lifespan alteration upon *SA1* depletion in developing neuroblasts are rescued by reduction of PARP activity.** (A) Schematics of NBII development and relative markers used to assess it. GMC, ganglion mother cells; INP, intermediate neural precursors; NB, neuroblasts. (B,C) Representative confocal images of NBII clusters of the indicated genotypes, labeled to detect the indicated differentiation markers. (D,E) Quantification of the indicated differentiation markers in NBII clusters of the indicated genotypes (D), or upon supplementation with vehicle (DMSO) alone or 2.5 μM 3-AB in vehicle (3-AB) (E). More than four brain lobes per sample were analyzed, representing a minimum of 22 NBII clusters, were used for the quantification. Samples were compared by one-way ANOVA, with uncorrected Dunn's multiple comparison test. Non-significant (ns), *P*>0.05; *P<0.05, **P<0.01, ***P<0.001, ****P<0.0001. In D, *P*-value intervals above the bars indicate comparisons with sample 1; lines indicate other meaningful comparisons. In E, *P*-value intervals above the bars indicate comparisons between treated and untreated samples. (F,G) Lifespan of animals of the indicated genotype fed with vehicle alone (DMSO; F) or 2.5 μM 3-AB in vehicle (G). Numbers in brackets indicate the day at which the population reached 50% survival. The experiment was repeated twice with *n*>250 animals per sample. Data of both experiments have been pooled and are shown as a single survival curve. Samples were pairwise compared with *NBII>luc-RNAi* controls using a Log-rank (Mantel–Cox) test. Non-significant (ns), *P*>0.05; *P<0.05, ****P<0.0001. *P*-value intervals in the key indicate comparisons with *NBII>luc-RNAi*. (H) A model of tumorigenesis based on reduced *SA1* expression and effects of PARP inhibition in such context.

The emergence of NSC-derived undifferentiated brain cells in *SA1*-depleted adults might result from altered differentiation of larval NBII clusters. To analyze their state at 120 AEL, we immunolocalized Miranda (Mira), a marker of the intermediate neural precursors (INPs) derived from stem cells; Prospero (Pros), a marker of ganglion mother cells (GMCs) that are produced by mature INPs; and Elav, which marks differentiated neurons in L3 larvae (Fig. 5A). In control *NBII>luc-RNAi* animals, we observed the expected distribution of Mira^+^, Pros^+^ and Elav^+^ cells emerging from GFP^+^ clusters. In sheer contrast, *NBII>SA1-RNAi* clusters accumulated Mira^+^ cells at the expense of Pros^+^ and Elav^+^ cells. Similar results were obtained in *SA1^ex86^/+* animals (Fig. 5B, quantified in Fig. 5D). These data suggest that reduced *SA1* expression delays the transition of INPs to GMCs.

We next tested whether NBII differentiation is altered by the reduction of *Parp1* activity. In *NBII>Parp1-RNAi* NBII clusters, we observed a slight increase in Mira^+^ cells but no change in Pros^+^ and Elav^+^ cells, compared to those in *NBII>luc-RNAi* controls (Fig. 5C, quantified in Fig. 5D), suggesting that decreased Parp1 activity causes only a minor alteration to NBII differentiation. Remarkably, downregulation of *Parp1* ameliorated the NBII differentiation defect observed in *SA1-*depleted or *SA1^ex86^/+* animals (Fig. 5C, quantified in Fig. 5D). To determine whether inactivation of Parp1 during NBII differentiation could also be achieved pharmacologically, we fed animals with 3-AB. Consistent with our genetic data, we observed that 3-AB supplementation did not affect NBII differentiation. However, it partially phenocopied the effects of *Parp1* depletion, with amelioration of Pros^+^ and Elav^+^ cell differentiation (Fig. 5E). These results suggest that SA1 supports NB cluster development and that the defects observed upon *SA1* reduction are rescued by impairment of PARP activity.

Despite the overall morphology of control, *SA1*-downregulated and *SA1* heterozygous animal adult brain appearing unaffected (Fig. S7), we observed that *NBII>SA1-RNAi* animals displayed a 17% reduction in median survival, compared to that of *NBII>luc-RNAi* animals. Similar data were obtained in control animals heterozygous for a null *SA1* allele (*NBII SA1^ex86^/+*; Fig. 5F). Consistent with the effects of 3-AB supplementation on NBII differentiation, although 3-AB supplementation did not significantly alter the lifespan of control *NBII>luc-RNAi* animals, it improved the lifespan of the animals with reduced *SA1* expression (Fig. 5G). The rescuing effect obtained by 3-AB supplementation was not observed in *NBII>brat-RNAi* animals (Fig. 5F,G).

Overall, we find that the reduction in PARP activity counteracts the tumor-associated effects of *SA1* downregulation.

## DISCUSSION

Database analyses highlighted a possible role of STAG2 in brain tumors, particularly in glioblastoma and medulloblastoma. As reported variants are predicted to result in STAG2 haploinsufficiency, we exploited an *in vitro* and *in vivo* system for modeling the contribution of reduced *STAG2* activity to relevant processes. Using HEK-293T cells with stable downregulation of *STAG2* as a human non-transformed 2D and 3D *in vitro* model, we first observed accumulation of DNA damage. Interestingly, when we exposed depleted cells to PARP inhibitors, we detected a statistically significant increase in cell death relative to baseline levels, suggesting the possibility of additive or synthetic lethal effects when both *STAG2* and PARP activity are reduced. To test the *in vivo* relevance of our *in vitro* data, we took advantage of pharmacogenetic manipulation of cohesin and PARP activity in *Drosophila melanogaster.* Based on depletion or heterozygosity of the fly *STAG2* homolog *SA1* and/or depletion of *Drosophila Parp1* (or inhibitor supplementation), we confirm the presence of DNA damage, as well as cell death in multiple tissues. We also reveal that cohesin genes and, in particular, *SA1* act as tumor suppressor in flies. In particular, we report, for the first time, that *SA1* depletion or heterozygosity is sufficient to delay, and possibly occasionally derail, terminal differentiation of brain stem cells and that *SA1* depletion promotes tissue growth in a model of gliomagenesis. Stinkingly, these tumor-relevant phenotypes are reverted by depletion or inhibition of PARP activity. We also find presence of cCas3 activation in most tissues analyzed upon *SA1* or *Parp1* (or their combined) depletion. Whether this correlation implies a causation remains to be determined (see model in Fig. 5H).

A recent study in *Drosophila* has found that alteration to epigenetic regulation of chromatin by impairing Polycomb activity is sufficient to promote tumorigenesis by changing genetic programs in the absence of driver mutations (Parreno et al., 2024). Consistent with this, we observe delays in NBII differentiation and spontaneous retention of stem cell-derived masses in the fly adult brains. Thus, the regulation of chromatin architecture could be an important function of cohesin proteins relevant to tumor suppression. In favor of this, we also observe that *SA1* depletion mostly impacts nuclear – and possibly cell – size during gliomagenesis. These alterations might well be the result of the concerted changes in gene expression typical of altered chromatin regulation. However, in fly tissues upon *SA1* downregulation, we also observe accumulation of DNA damage, formation of defective mitotic figures and apoptosis, together suggesting that the effects of SA1 impairment are pleiotropic. Separation of function mutations will be required to attach to each cohesin function the correct tumorigenic potential. Interestingly, in different tissues, we observe slightly different effects of reduction of *SA1* expression and quantitatively different interactions with PARP inactivation, indicating that the effects of cohesin loss and PARP activity are highly context dependent.

In NSCs, we conclude that correct *SA1* expression supports NB differentiation during larval life and prevents the persistence of NBII-derived masses. Although these masses in adult brains are occasional, and we have not yet characterized their nature, cohesin genes have been implicated in axonal pruning (Schuldiner et al., 2008), suggesting defects in post-mitotic elimination of persistent NBIIs. *SA1* depletion has also recently been shown to increase the migration of tumor cells of epithelial origin in flies (Canales Coutiño et al., 2020). Thus, it will be interesting to also study whether the brain masses observed in our experiments have migrated away from sites of NBII development.

Precise control of NB cell elimination during embryonic nervous system development depends on *SA1* activity, downstream of H3K27me3 chromatin state modulation by Notch (Arya et al., 2019). Remarkably, epigenetic regulation by Polycomb proteins increases the activity of stemness genes during asymmetric cell division of NBII by elevating H3K27me3 levels at cis-regulatory elements in INP cells. These authors suggest that failure of this process could reduce Notch activity and thereby promote INP proliferation instead of maintaining their stemness (Rajan et al., 2023). In this context, Notch activity is also known to be repressed by the tumor suppressor Brat (human TRIM3) to promote differentiation of immature neural precursors (Hadjipanayis and Brat, 2017). Consistent with this, the masses observed in adult fly brain with reduced NSC expression of *SA1* resemble those more abundantly obtained upon *brat* depletion. Thus, it would be interesting in the future to assess whether *SA1* reduction alters Notch regulation of NBII development.

How do the differentiative phenotypes discussed above correlate with DNA damage and cell death? At present, our correlative data do not clarify whether the accumulation of DNA damage and activation of cell death contribute to or hinder tumorigenesis. However, such phenotypes are reminiscent of those reported in response to replication stress upon cohesin removal, or PARP inhibition, or oncogenic MYC activity (Colicchia et al., 2017; Benedict et al., 2020; Peripolli et al., 2024). Consistent with this, we also observed the occasional presence of persistent masses derived from NBII cells in fly brain overexpressing NMYC. In epithelial tissue, PARP inhibition is sufficient to cause accumulation of DNA damage; in addition, the combination of PARP inhibition and reduction of *SA1* expression results in a synergistic effect. However, *in vivo*, we do not observe the synthetic lethality suggested by *in vitro* experiments. This discrepancy might depend on the complex relations entertained by cells in a tissue context. Despite this, our *in vitro* and *in vivo* results are in line with reports that glioblastoma cells with *STAG2* variants show an increase in DNA damage markers and cell cycle arrest caused by replication stress when treated with PARP inhibitors (McLellan et al., 2012; Tothova et al., 2021; Bailey et al., 2014), and with a clinical trial (NCT03974217) exploring PARP inhibition in blood cancers with variants in cohesin genes. Moreover, they suggest that the radiation sensitivity observed upon PARP inhibition in *in vitro* models of certain types of medulloblastoma (Price and Lau, 2023) might involve cohesin activity.

Overall, we have laid the groundwork for future in-depth analysis of the combined effects of reduced cohesin activity and PARP inhibition in a practical, inexpensive and 3R (Replacement, Reduction and Refinement)-compliant set of *in vivo* models of brain development and tumorigenesis. Further study of these models will yield an understanding of how to harness PARP inhibition to revert the effects of reduced cohesin activity in cancer.

## MATERIALS AND METHODS

### Cell cultures and cell-based assays

HEK-293T cells were grown in Dulbecco's modified Eagle's medium (DMEM; Life Technologies, 11965092) supplemented with 10% fetal bovine serum (FBS; Life Technologies, 10500064) and 1% penicillin-streptomycin (P/S; Euroclone, ECB3001D). Cells were cultured in a Petri dish at 5% CO_2_ and 37°C.

HEK-293T shSCR and HEK-293T shSTAG2 are derived from a HEK-293T control cell line obtained through viral infection with a control scrambled (SCR) plasmid and one containing a short hairpin (sh) for *STAG2* gene silencing (shSTAG2); both plasmids also contain a GFP-encoding gene and puromycin cassette for selection. Briefly, STAG2-shRNA lentiviral vector (Origene, 10735) or the control SCR sequence SCR-shRNA, as well as viral packaging and envelope components, was transfected into HEK-293T cells using CaCl_2_ method. After 2 days, media containing virus particles were collected and used to infect new HEK-293T cells to generate stable cell lines. Cells were positively selected with puromycin (1 μg/ml Invivogen ANT-PR-5) treatment for 72 h from 48 h post-infection and then periodically maintained under selection.

To perform 3-(4,5-dimethylthiazol-2-yl)-2,5-diphenyltetrazolium bromide (MTT) assays, 20,000 cells/well were seeded in a 24-well multiwell until 70% confluence. Then, the culture medium was removed, and 300 µl serum-free blank medium and 30 µl MTT stock solution (5 mg/ml MTT powder; Sigma-Aldrich, M2003) were added to each well. The plate was incubated at 37°C for 30 min/2 h until purple precipitates appeared. After removing the solution, 300 μl isopropanol was added to each well, and the plate was shaken for 10 min at room temperature (RT). After resuspension, 200 µl from each well was transferred into a 96-well multiwell with lid. The levels of precipitates were read by an Ensight plate reader (PerkinElmer) at 570 nm.

For adhesion tests, cells in triplicates were seeded at a concentration of 40,000 cells per well in a 24-well plate containing a glass slide, then the plate was incubated for 2 h at 37°C. After medium removal, cells were fixed with 300 µl cold methanol for 10 min at −20°C. After fixation, each well was washed twice with 1× PBS for 5 min and 300 µl Hematoxylin was added for 1 min at RT. After Hematoxylin removal, each well was washed with water until it became clear, and 300 µl Eosin was added for 1 min at RT. Each well was washed with water, and slides were mounted on a slide with Mowiol.

To assess spheroid formation, 5000 cells/well were seeded in a low attachment 96-well U-bottom multiwell. Cells were resuspended in DMEM/F12 medium (Gibco, 11320033) containing B-27 supplement (100×; Gibco, 12587010), N-2 supplement (50×; Gibco, 17502048), heparin (2 μg/ml; Merck Life Science, H3149-50KU), EGF (20 ng/ml; Prepotech, AF-100-15-500UG), FGF2 (10 ng/ml; Prepotech, AF-100-18B-500UG) and 100× P/S. Then, 200 μl/well was aliquoted, and the plate was centrifuged for 5 min at 300 ***g***. Spheroids of each cell line were grown for 4 days and then moved to a 24-well plate previously coated with Matrigel (Merck Life Science, CLS356234-1EA) to evaluate dissemination ability.

For PARP inhibition, shSCR and shSTAG2 HEK-293T cells and 4-day-old spheroids were treated with 3-AB (Selleck USA, S1132) at 500 µM for 72 h and then fixed for further experiments. An equal volume of DMSO was used as a vehicle control.

TUNEL assay was performed on fixed HEK-293T cells using an *in situ* cell death detection AP kit (Roche 11684809910) according to the manufacturer's instructions. Slides were mounted with Mowiol, and images were acquired on a LEITZ DMRB (Leica) microscope.

### *Drosophila* methods

*Drosophila* strains were maintained in vials containing a standard food medium composed of water, 34% cornmeal, 57% molasses, 9% yeast, 0.7% agar, 0.7% propionic acid and 2% Tegosept (Apex, 99763). Supplemented food composition was modified by replacing molasses with 35% sucrose and with 44% yeast concentration to enhance egg laying. Unless otherwise specified, fly lines were generated by crossing or recombination from stocks obtained in our laboratory or sourced from the Bloomington *Drosophila* Stock Center (BDSC), Indiana University and the Vienna *Drosophila* Resource Center (VDRC). Crosses were kept at 25°C. The fly stocks used are provided in Table S2.

For *in vivo* PARP inhibitor supplementation, 3-AB (Selleck USA, S1132) was used as PARP inhibitor. To establish an effective and non-toxic dose, a 50 mM stock solution was prepared in DMSO following the manufacturer's datasheet and diluted in sterile distilled water to concentrations of 5-250 μM. 150 μl of each of these concentrations was added to a culturing tube containing 3 ml *Drosophila* white food, pre-punctured with 20 holes for uniform diffusion. Equal volumes of DMSO diluted in distilled water served as controls. For all other treatments, animals were reared in tubes containing food supplemented with a final concentration of 2.5 μM 3-AB or vehicle (DMSO). In lifespan experiments, cohorts of 20 flies/genotype (ten males and ten females) per vial were kept at 25°C. Flies were transferred to fresh food medium every 2-3 days, when dead flies were also scored.

In gliomagenesis and NB experiments, animals were synchronized by short times of egg deposition and larvae were collected at 120 h AEL, dissected and processed for immunofluorescence. For estimation of pupal lethality, tubes previously cleared of parentals after egg deposition were analyzed after 25 days of culturing. At the time of analysis, all viable offspring were removed, and the dead animals found in the tube were categorized according to their appearance: animals dead before pupariation that have crawled up the tube or that are visible as L3 in the food (L3 lethality); dead animals that have pupariated in which no metamorphosis has occurred (early pupa), which appear as pupal cases that have not eclosed filled with dried clear tissue; and dead animals that have pupariated and that present signs of metamorphosis, such as blackened eyes or wings (late pupa).

Adult brains were dissected from 7- to 10-day-old animals and processed for immunofluorescence.

### Real-time quantitative PCR

For cell culture experiments, 1 µg RNA extracted with phenol/chloroform method by NucleoZOL (Macherey-Nagel) was retrotranscribed by using an All-In-One 5X RT MasterMix (Microtech) kit. For real-time quantitative PCR (RT-qPCR), a TB Green Premix Ex Taq (Tli RNase H Plus) (Takara) kit and the CFX Opus 96 Real-Time PCR System (Bio-Rad) were used to evaluate gene expression following manufacturer instructions. The data obtained from RT-qPCR were analyzed using a comparative Ct quantification method. ΔCt was obtained by normalizing each Ct sample to the mean of the housekeeping genes (*GAPDH*, *RPLP0*, *RPL13A*). Then the ΔΔCt was obtained by comparing the ΔCt of every sample for each gene to the reference one for gene expression of the treated samples against their control. Relative gene expression values were obtained by calculating the fold change, which corresponds to 2^−ΔΔCT^. Technical and biological triplicates were performed for all the experiments. The following primers were used for RT-qPCR: *GAPDH* (For, 5′-AGCCACATCGCTCAGACAC-3′; Rev, 5′-GCCCAATACGACCAAATCC-3′); *RPLP0* (For, 5′-TCTACAACCCTGAAGTGCTTGAT-3′; Rev, 5′-CAATCTGCAGACAGACACTGG-3′); *RPL13A* (For, 5′-CCTGGAGGAGAAGAGGAAAGAGA-3′; Rev, 5′-TTGAGGACCTCTGTGTATTTGTCAA-3′); *STAG2* (For, 5′-AAGGAGGACTTGCTGCGTTT-3′; Rev, 5′-TCCTCTTGCTGACCATCTGC-3′). Analyses of RT-qPCR and western blot data were performed using Student’s *t*-test.

For *in vivo* experiments, after dissection, organs of the selected genotype were homogenized in Trizol reagent (Invitrogen, 15596-018). For wing disc analyses, 30 discs were used.

RNA extraction was performed using a commercial kit (Zymo Research RNA extraction kit for insect tissue). The concentrations of extracted RNA and DNA were measured using a NanoDrop 1000 Spectrophotometer (Thermo Fisher Scientific). Retrotranscription of RNA to cDNA was performed using a LunaScript^®^ RT SuperMix Kit (New England BioLabs, E3010). RT-qPCR was performed with Luna^®^ Universal qPCR Master Mix (New England BioLabs, M3003) by using a CFX Connect Real Time PCR Detection System (Bio-Rad, 1855201).

Results were normalized using the housekeeping *Rp49* and the ΔΔ cycle threshold method, and results were expressed as the relative change (-fold) of the downregulated group over the control group, which was used as a calibrator.

RT-qPCR for *SA1* and *Rp49* was performed using Sybergreen (Applied Biosystems) with the following primers: *SA1* (F, 5′-TTGTGCGACACTCGAAGAAC-3′; R, 5′-CCGCTTTCTTCGTCAAACTC-3′); *Rp49* (F, 5′-ACGTTGTGCACCAGGAACTT-3′; R, 5′-TACAGGCCCAAGATCGTGAA-3′).

Output data were analyzed using CFX Manager Software (Bio-Rad) and Prism, which was also used to prepare graphs.

### Protein extraction and quantification and western blot analysis

For cell extracts, HEK-293T pellets were resuspended in cold S300 buffer (300 mM NaCl, 50 mM HEPES pH 7.6, 0.1% NP40, 2 mM MgCl_2_, 10% glycerol) added with protease and phosphatase inhibitors and GENIUSTM Nuclease (SC-202391). Samples were left on ice for 1 h and then centrifuged at maximum speed (9000 ***g***) for 10 min at 4°C. Supernatants containing protein lysates were collected and quantified using Bradford method. 0.2 mg/ml bovine serum albumin (BSA; Bio-Rad 5000206) was used to prepare the standard curve. Finally, samples were predisposed for the western blot run by preparing aliquots at 1 µg/µl supplemented with 4× Laemmli sample buffer (LSB; Bio-Rad, 1610747) and boiled for 10 min at 100°C. 30 µg of samples were loaded and separated by SDS-PAGE (1× running buffer diluted from 10×, made of 3% Tris HCl, 14.4% glycine and 1% SDS) using 10% polyacrylamide gels (Mini-PROTEAN TGX Gels; Bio-Rad, 4561034). At the end of the run, 1× cold transfer buffer (20% methanol and 10% 10× transfer buffer, composed of 3% Tris HCl and 14.4% Glycine) was used to transfer protein samples to a nitrocellulose membrane (Merck Life Science, GE10600003). 5% milk (Sigma-Aldrich, 4259001) in 1× TBS-T (3% Tris-HCl, 8.7% NaCl and 0.2% KCl supplemented with 0.1% Tween 20) was used as a blocking solution for 1 h at RT, then membranes were incubated with primary antibody diluted in milk or 1× TBS-T at 4°C overnight (rabbit anti-STAG2, 1:1000, Cell Signaling Technology, 5882; rabbit anti-γH2AX, 1:1000 Cell Signaling Technology, BK9718S; rabbit anti-GAPDH, 1:1000, Cell Signaling Technology, 5174). Then, membranes were incubated with anti-rabbit or anti-mouse horseradish peroxidase-conjugated secondary antibody [1:3000, Bio-Rad, 1706515 (rabbit), 1706516 (mouse)] diluted in 1× TBS-T or in milk for 1 h at RT. Membranes were washed three times with 1× TBS-T and incubated with ECL or Amersham to detect chemiluminescence signals captured by a Chemidoc Imaging System.

### Immunofluorescence analyses

Cells were permeabilized for 10 min with PBT (PBS with 0.25% Triton X-100), and then natural donkey serum (NDS) (20% NDS in 0.25% PBT) was used as blocking solution for 1 h at RT. Cells were incubated with primary antibody rabbit anti-γH2AX (1:200, Cell Signaling Technology, BK9718S), and rabbit anti-cCas3 (1:200, Cell Signaling Technology, BK9664S) at 4°C overnight. The following day, secondary antibody donkey Alexa Fluor Cy-3-conjugated anti-rabbit Fab fragments (1:200; Jackson ImmunoResearch) was used and incubated for 2 h at RT. Then, cells were washed with 0.25% PBT three times, and DAPI (1:1000) was used for nuclei counterstaining. Finally, cells were washed with 1× PBS and with MilliQ water. Slides were mounted with Mowiol (Sigma-Aldrich), and signals were acquired by fluorescence microscope at 10× magnification and, for cCas3, quantified by ImageJ software.

For immunolabeling of *Drosophila* organs, adult brain were dissected and processed as described (Wu and Luo, 2006; Ostrovsky et al., 2013). Larvae were reared for 120-150 h post-egg deposition, and wandering third-instar larvae were selected for analysis. Larval brains and wing discs remained attached to carcasses for ease of handling. Carcasses were prepared by removing the gut, fat tissue and salivary glands in 1× PBS, then fixed in 4% paraformaldehyde for 20 min at RT. Tissues were rinsed three times in 0.1% Triton X-100 in 1× PBS (PBST) for 5 min to remove fixative, followed by permeabilization in 0.3% Triton X-100 in 1× PBS (0.3% PBST) for 30 min. Blocking was performed with 5% BSA in 0.3% PBST for 30 min before overnight incubation with primary antibodies diluted in blocking solution. The following primary antibodies were used: chicken anti-GFP (1:1000, Abcam, ab92456), rabbit anti-Mira (1:500, Abcam, ab197788), mouse anti-Pros [1:100, Developmental Studies Hybridoma Bank (DSHB), 528440], rat anti-Elav (1:50, DSHB, 528218), mouse anti-histone 2A gamma variant, phosphorylated (γH2Av) (1:50, DSHB, 2618077), rabbit anti-Fibrillarin (1:500, Abcam, ab5821), mouse anti-nc82 (also known as Brp; 1:40, DSHB, 2314866), rabbit anti-cCas3 (1:200, Cell Signaling Technology, 9661), mouse anti-pH3 (1:2000, Abcam, ab14955) and mouse anti-Repo (1:200, DSHB, 528448). After three washes, Alexa Fluor-conjugated secondary antibodies (1:300, Invitrogen) were incubated for 2 h at RT. DNA was stained with DAPI (1:5000, Sigma-Aldrich), followed by three washes and fine dissection. Samples were mounted in Mowiol (Sigma-Aldrich) and dried overnight at RT.

### Microscope acquisition

Brightfield images of 2D cell plates were acquired at 40× magnification, and images of 3D spheroids were acquired at 4× magnification. Confocal images were acquired with a Nikon A1R/AX laser scanning confocal microscope equipped with a Nikon A1/AX plus camera and the following objectives (Nikon): Plan Fluor 10× DIC L N1 (0.3 NA), Plan Fluor 20× DIC N2 (0.5 NA). DAPI and Alexa Fluor Cy-3 were excited at 405 and 561 nm, respectively, and observed at 425-475 and 570-620 nm, respectively.

Images of *Drosophila* organs were acquired using Nikon A1-SIM or NiU confocal microscopes from the UniTECH NOLIMITS departmental platform at varied magnifications. Immunofluorescence images were analyzed using FIJI (Fiji is just ImageJ, National Institutes of Health), followed by statistical analysis with Prism (version 9.1.2; GraphPad Software). Measurements and fluorescence evaluation were carried out through the FIJI software. Quantification analysis was performed by evaluating equal numbers of *z*-stack among different genotypes for comparative analysis.

### Database searches

The publicly accessible cBioPortal for cancer genomics database was investigated, selecting datasets from eight studies of medulloblastoma (Jones et al., 2012; Pugh et al., 2012; Robinson et al., 2012; Morrissy et al., 2016; Northcott et al., 2017) and glioblastoma (Brennan et al., 2013; Zhao et al., 2019; Wang et al., 2021). The CADD score, a numerical ranking that predicts the deleteriousness of a protein variant, was calculated using CADD v1.7 for all single-nucleotide polymorphism *STAG2* variants reported in gnomAD and the ones identified in cBioPortal. Patients belonging to two different datasets were considered only one time.

### Quantifications

All cell culture experiments were performed in biological and technical triplicates. To quantify the cCas3 signal, the ImageJ software and the integrated raw density (IntDen) method was used (Shihan et al., 2021). We counted four different fields, and the integrated density for every field was calculated with an adjusted threshold of 183. For adhesion tests, three images for each well for each condition were acquired, and cells were counted by ImageJ software. Then, the average of each replicate was normalized to the average of control cells for each experiment. Data obtained from western blots were analyzed using ImageJ software, and the mean pixel intensity was calculated. In the TUNEL experiment, at least three images for each condition for each experiment were acquired. Cells were counted by ImageJ, and the ratio of TUNEL-positive cells over the total cells in a field was calculated.

In fly experiments, for the analysis of larval wing discs acquired at 20× magnification, a selection mask was created around the GFP^+^ tissue to quantify surface area. For gliomagenesis analyses, the total brain area was quantified by drawing a region of interest (ROI) on the maximum projection of the entire brain (30-35 *z*-slices). For mid-section analysis, comparable single-plane images were acquired, the optic lobe area was selected with ‘Freehand selection’, and the percentage of fluorescence per channel was measured by thresholding. For Repo/DAPI analysis, the intensity ratio between the two channels was calculated within the same selection. The number of Repo^+^ cells per lobe was obtained using the Cell Counter 2D plugin (Fiji). Repo^+^ nuclear areas were manually drawn (‘Freehand selection’) in 20 cells per optic lobe and reported as mean value. For the measurements of GFP^+^ cell masses in adult brains, the GFP^+^ area was measured on a maximum projection of the full *z*-stack using FIJI's ‘Freehand selection’ tool and quantified with the ‘Measure’ command. For immunofluorescence analysis of NBII a ten-slice *z*-stack (0.2 μm per slice) was acquired for each sample. The GFP^+^ outer contour was outlined using the ‘Freehand selection’ tool, creating an ROI mask applied to individual fluorescence channels. The ‘Threshold’ function defined positive signals relative to background, and the occupied area was measured as the percentage of pixels within the ROI.

All counts were analyzed with GraphPad Prism software. The selected statistical test and sample size are shown in the figures or in figure legends. In all analyses, *P*<0.05 was considered significant. Each sample was compared to sample 1 (control), and *P*-value intervals are reported above the value, unless otherwise noted. Non-significant comparisons are not indicated, unless when relevant. Other meaningful comparisons and relative *P*-value intervals are indicated with lines above the values connecting the samples compared.

## Supplementary Material

10.1242/dmm.052440_sup1 Supplementary information

Table S1. Patient data.
