# Supplementary material for: Tumor-suppressive activities of SA1/STAG2 and effects of PARP impairment during brain development
Source: Dis Model Mech. 2026 Feb 2;19(1):dmm052440. doi: 10.1242/dmm.052440 (PMC12919954; doi:10.1242/dmm.052440)
Supplement: Supplementary information [file dmm-19-052440-s1.pdf]

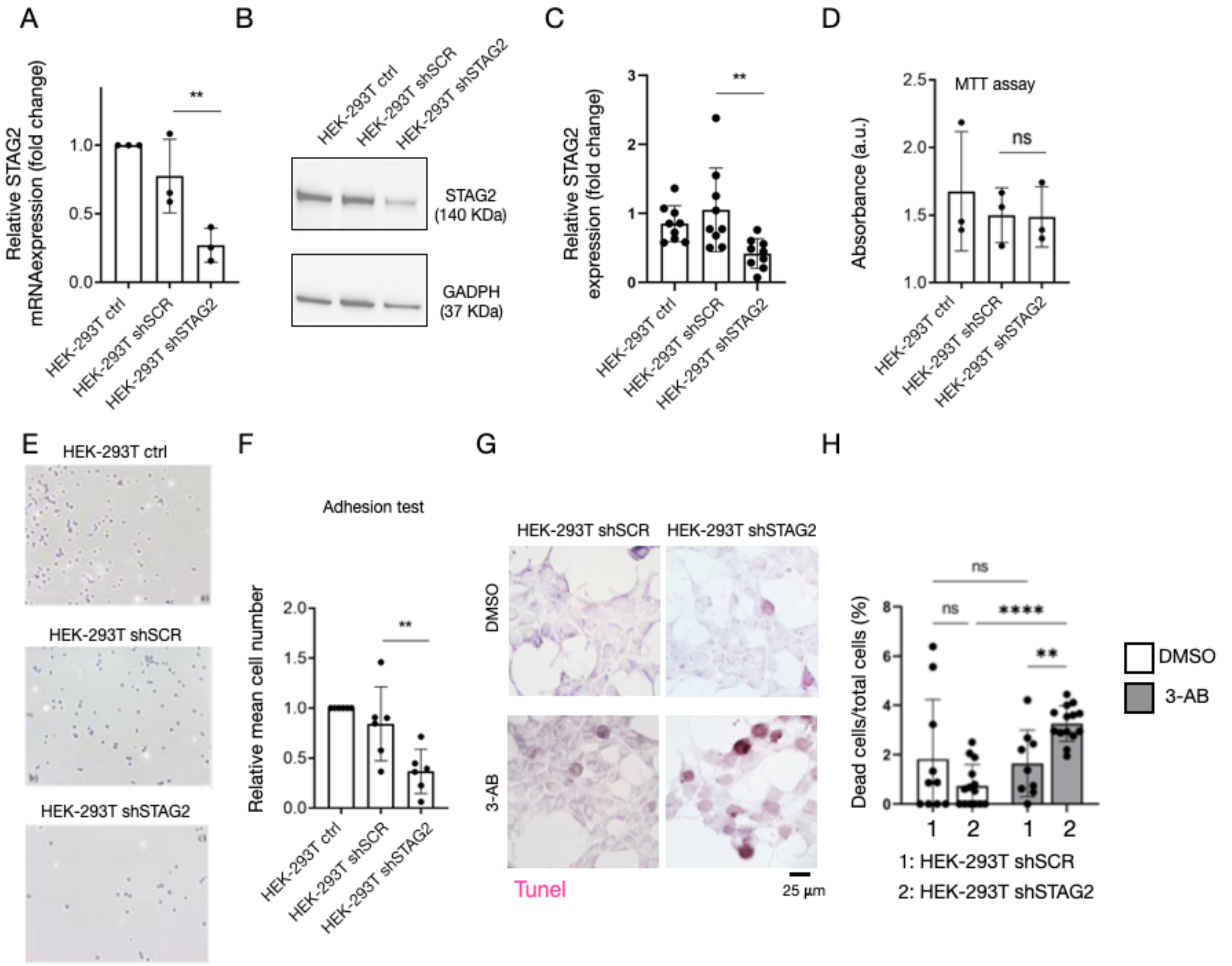

Fig. S1. *STAG2* depletion affects cell adhesion. (A) RT-PCR quantification of *STAG2* mRNA expression in control cells or cells expressing the indicated shRNA. Each data point represents a biological replicate. shSTAG2 and shSCR samples were compared by one-way ANOVA, with uncorrected Fisher's LSD test. **(**B-C) Western blot analysis of STAG2 and GADPH expression in control cells or cells expressing the indicated hairpin. The blot of GADPH to normalize is the same of Fig. 1C. Each data point represents a technical replicate across 3 biological replicates. shSTAG2 and shSCR samples were compared by one-way ANOVA, with uncorrected Dunnett's multiple comparison test. (D) Determination of cell density of control cells or cells expressing the indicated hairpin. Each data point represents a technical replicate. shSTAG2 and shSCR samples were compared by one-way ANOVA, with uncorrected Fisher's LSD test. (E-F) Substrate adhesion of control cells or cells expressing the indicated shRNA and relative quantification. shSTAG2 and shSCR samples were compared by one-way ANOVA, with uncorrected Fisher's LSD test. (G-H) Representative brightfield

Disease Models & Mechanisms • Supplementary information

images of the indicated HEK-293T cell lines treated with DMSO or 3-AB at a concentration of 500uM, and relative quantification (H). The percentage of TUNEL+ cells per sample is illustrated. Each data point represents a technical replicate across 3 biological replicates. the The 3-AB HEK-293T shSTAG2 sample was compared to HEK-293T shSTAG2 treated with DMSO or to HEK-293T shSTAG2 shSCR treated with 3-AB by one-way ANOVA, with uncorrected Fisher's LSD test.

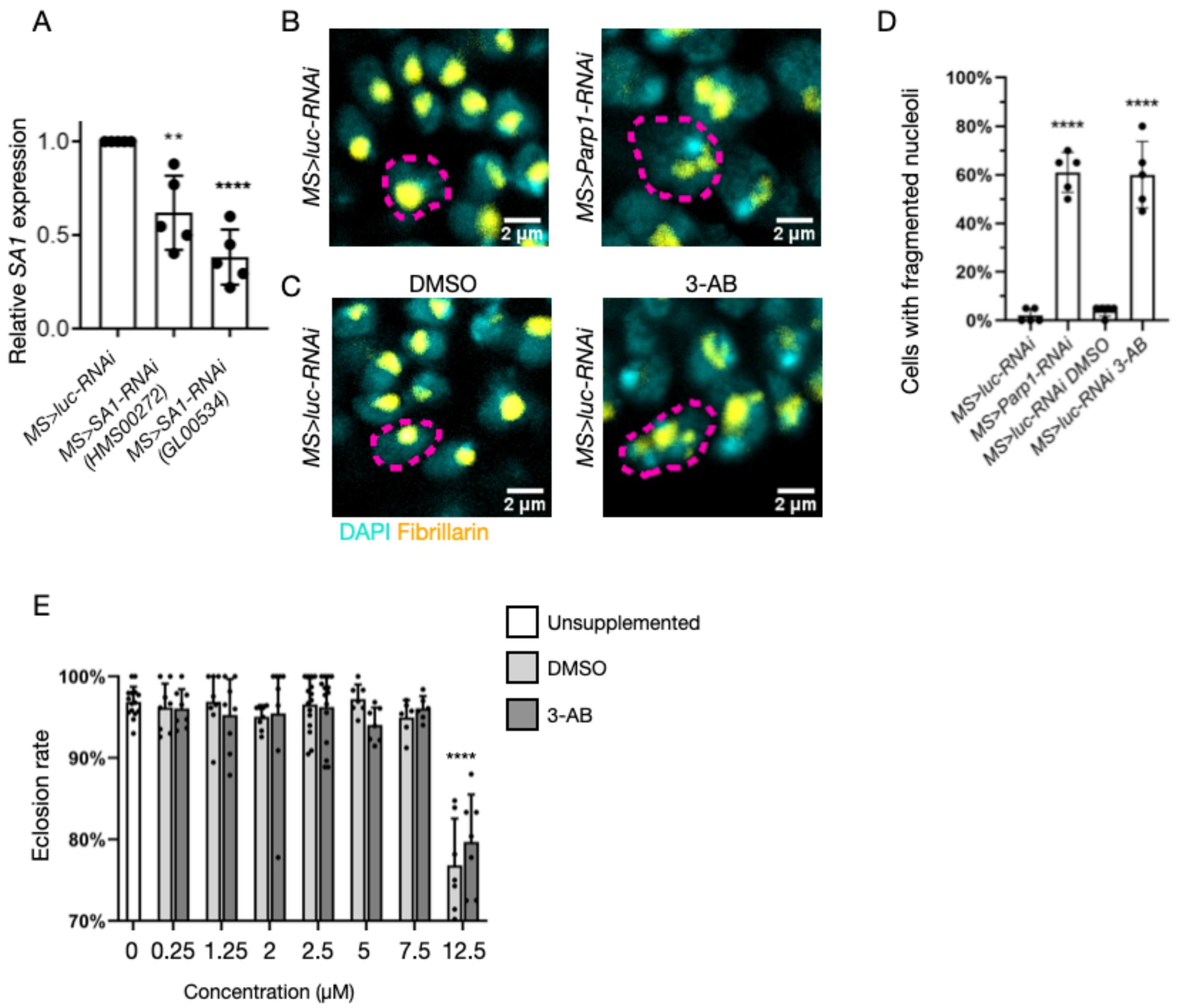

Fig. S2. *SA1* downregulation and reduction of PARP activity. (A) RT-PCR quantification of *SA1* mRNA expression in extracts of wing imaginal discs of the indicated genotypes. Each data point represents extracts of 30 wing discs. Samples were compared to *MS>luc-RNAi* controls by one-way ANOVA, with uncorrected Fisher's LSD. (B-C) High magnification single section confocal images of nuclei of the wing pouch of animals of the indicated genotypes treated with vehicle (DMSO) or 2.5 µM 3-AB in vehicle (3-AB), that have been labeled to detect the DNA (DAPI) and the nucleolar marker fibrillarin. Example nuclei are enclosed by dashed lines. (D) Quantification of nucleolar fragmentation. Each data point represents a single disc in which 20 nuclei scored for fragmentation. N=5 discs. Samples were compared to controls by unpaired t-test. (E) Eclosion rates of control animals untreated or fed with the indicated concentration of vehicle alone (DMSO) or 3-AB in vehicle (3-AB). Each data point represents 1 fly vial. N>7 vials analyzed. Mock-treated and treated samples were compared by two-way ANOVA, with uncorrected Fisher's LSD.

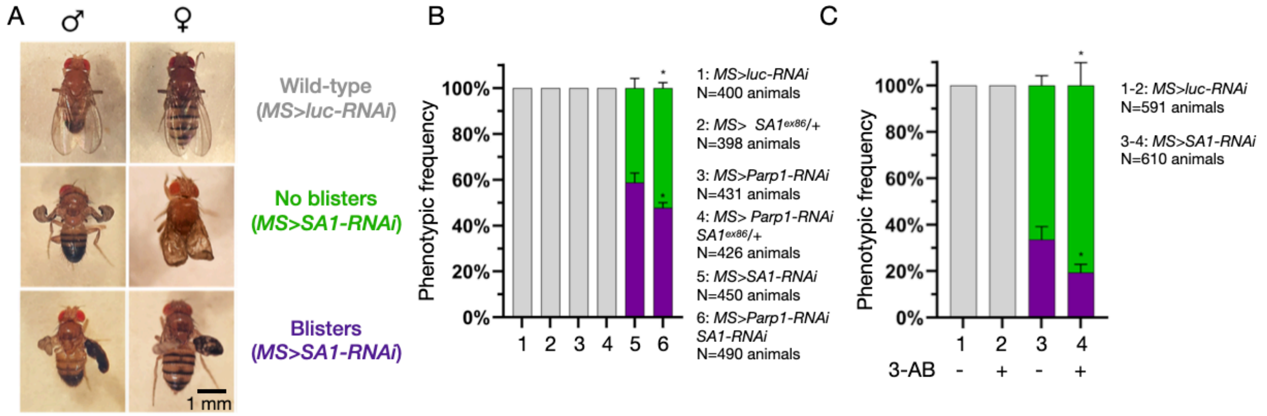

Fig. S3. Amelioration of *SA1* depletion phenotypes upon reduction of PARP activity. (A) Classification of wing phenotypes of control animals or animals with *SA1* downmodulation. (B-C) Quantification of the wing phenotypes of animals of the indicated genotypes and treated as indicated. In C, 3-AB was used at 2.5 µM in the food. The number of analyzed animals of each condition is >400. Samples were compared by two-way ANOVA, with Šídák's multiple comparisons test. P- values intervals above the bars indicate comparisons with sample 5 (B) or 3 (C).

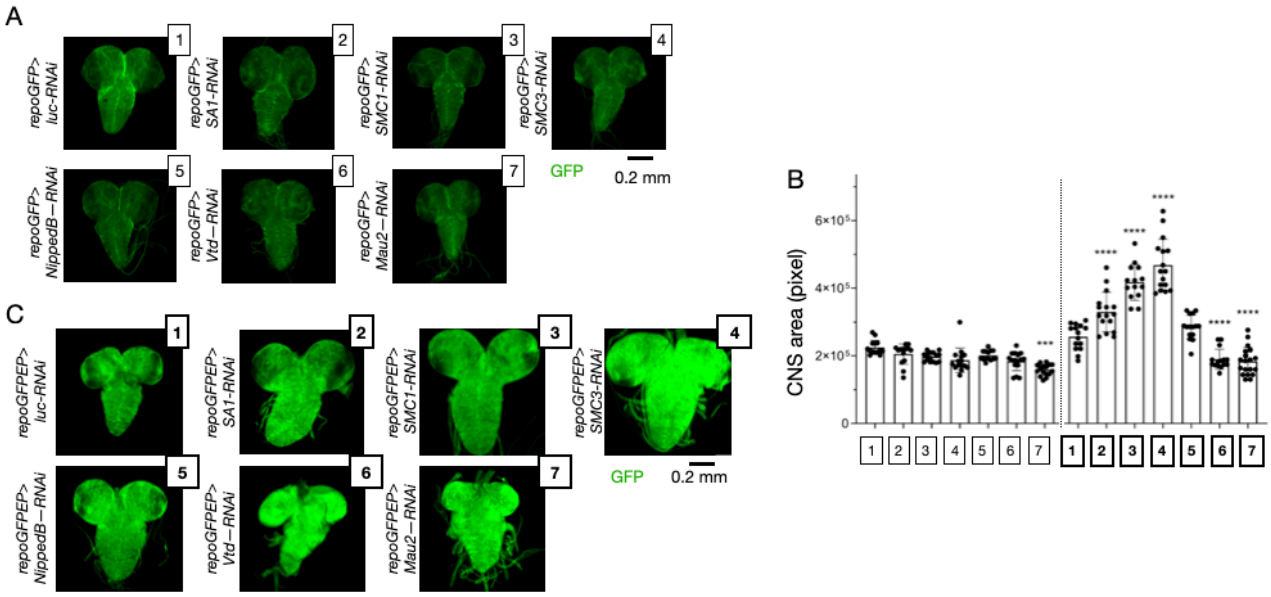

Fig. S4. Cohesin complex depletion during gliomagenesis. (A-C) Maximum projection of confocal z-stack spanning the entire larval CNS of animals of the indicated genotype (A, C) and relative quantification (B). In B, the area of >12 CNS per sample is indicated. In both graphs, samples were all compared to control (sample 1) by one-way ANOVA, with Šídák's multiple comparisons test.

Disease Models & Mechanisms • Supplementary information

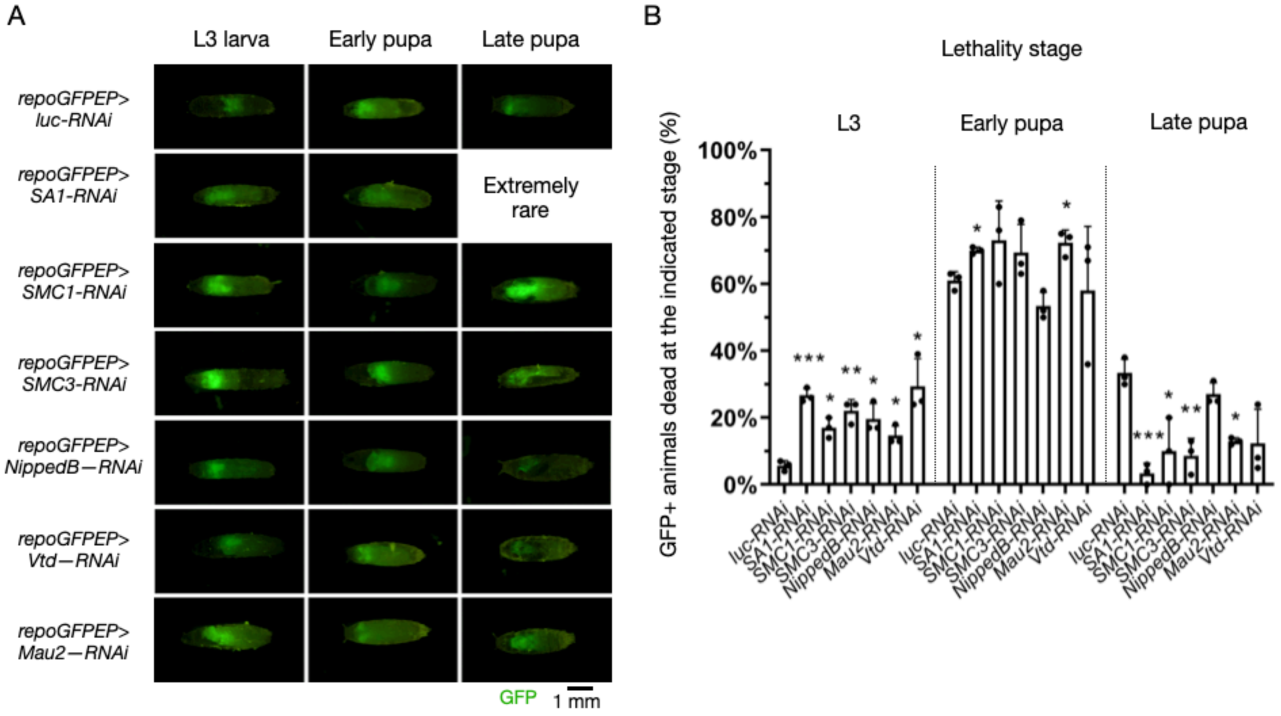

Fig. S5. Lethality stage of animals with reduced cohesin gene expression. (A-B) Representative fluorescence images of *repo>GFPEP* larvae depleted of the indicated cohesin gene and quantification of their lethality stage. The average of 3 experiments is presented with>30 animals per sample. Samples were compared to luc-RNAi controls by two-way ANOVA, with uncorrected Fisher's LSD.

Disease Models & Mechanisms • Supplementary information

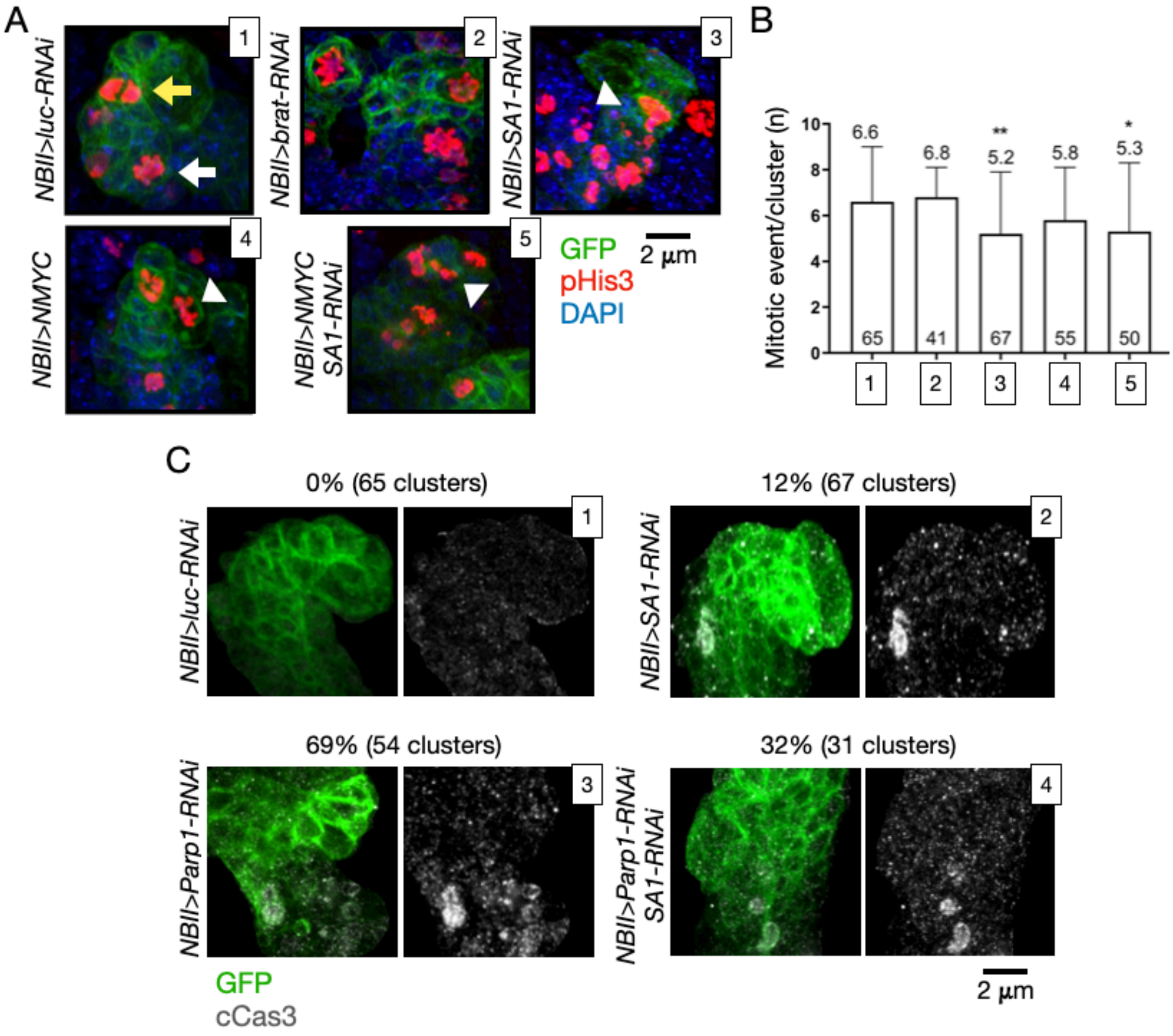

Fig. S6. Profiling of NBII clusters. (A-B) Representative single section confocal images of NBII clusters of the indicated genotypes, labeled to detect the cell proliferation marker pHis3 and relative quantification (B). In A, the yellow and white arrow point to a normal anaphase and prophase, respectively, while the arrowhead points to examples of aberrant mitotic features. In B, the number of clusters analyzed for each sample is listed at the base of each bar. Samples were all compared to control (sample 1) by one-way ANOVA, Dunnett's multiple comparisons test. (C) Representative maximal projections of a z-stack of confocal section of NBII clusters of the indicated genotypes, labeled to detect the apoptosis marker cCas3 and relative quantification of the frequency of cCas3+ cells and number of clusters analyzed. Please note that the average number of cCas3+ cells in the clusters that are found cCas3+ is of 1 in sample 2, 2.7 in sample 3 and 1.4 in sample 4.

Disease Models & Mechanisms • Supplementary information

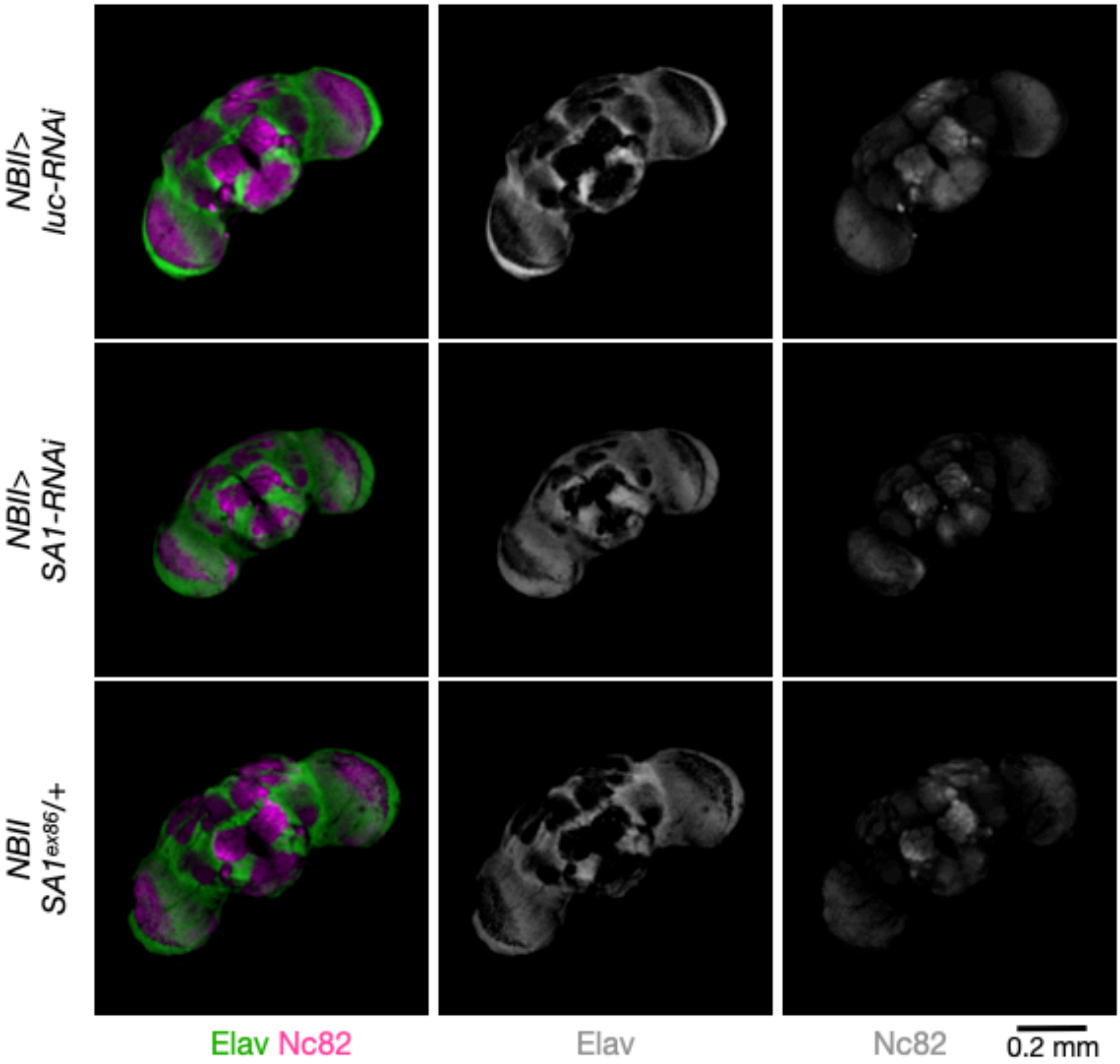

Fig. S7. Brain morphology with differing levels of SA1 expression. Confocal images of dissected adult brains of the indicated genotype, immunolabeled to detect the indicated protein markers. Nc82 marks the neuropils, while Elav marks the nucleus of neurons allowing to discriminate the adult brain morphology.

Table S1. Patient data.

Available for download at
https://journals.biologists.com/dmm/article-lookup/doi/10.1242/dmm.052440#supplementary-data

Disease Models & Mechanisms • Supplementary information

Table S2. Fly stocks used.

Code	Origin	Genotype
64349	BDSC	*CantonS*
31603	BDSC	*y[1] v[1];+; P{y[+t7.7] v[+t1.8]=TRiP.JF01355}attP2*
	This Work	*w118, ms1096Bx-GAL4,P{w[+mC]=UAS-EGFP}8; +; +*
	Cédric Maurange, Aix Marseille University	*UAS-dicer2;wor-GAL4, ase-GAL80/;UAS-mCD8::GFP*
36794	BDSC	*y[1] sc[*] v[1] sev[21]; P{UAS-TRiP.GL00534}attP40/CyO,Tb; +*
33395	BDSC	*y[1] sc[*] v[1] sev[21]; +; P{y[+t7.7] v[+t1.8]=TRiP.HMS00272}attP2*
	McKim, The State University of New Jersey	*+, SA[ex86] pr FRT40A / CyO; +*
	This Work	*+; P{UAS-TRiP.GL00534}attP40/CyO,Tb;P{GD9445}v46745*
	This Work	*+; SA[ex86] pr FRT40A/CyO,Tb;P{GD9445}v46745*
46745	VDRC	*w1118;+;P{GD9445}v46745*
	Renee Read, Emory University	*UAS DP110-CAAX; +; Repo GAL4-UASmCD8GFP,UAS ΔEGFRλ/TM6c,Tub-GAL80*
	Renee Read, Emory University	*+; +; Repo GAL4-UASmCD8GFP/TM2*
65229	BDSC	*y[1] sc[*] v[1] sev[21];P{y[+t7.7] v[+t1.8]=TRiP.HMC06090}attP40; +*
36598	BDSC	*y[1] sc[*] v[1] sev[21]; +; P{y[+t7.7] v[+t1.8]=TRiP.GL00558}attP2*
36783	BDSC	*y[1] sc[*] v[1] sev[21]; P{y[+t7.7] v[+t1.8]=TRiP.GL00518}attP40; +*
36794	BDSC	*y[1] sc[*] v[1] sev[21]; P{TRiP.GL00534}attP40 / CyO; +*
41976	BDSC	*y[1] sc[*] v[1] sev[21]; +; P{y[+t7.7] v[+t1.8]=TRiP.HMS02374}attP2*

Disease Models & Mechanisms • Supplementary information
